# Differential color development and response to light deprivation of fig (*Ficus carica* L.) syconia peel and female flower tissues: transcriptome elucidation

**DOI:** 10.1186/s12870-019-1816-9

**Published:** 2019-05-23

**Authors:** Ziran Wang, Miaoyu Song, Yunze Li, Shangwu Chen, Huiqin Ma

**Affiliations:** 1 College of Horticulture, China Agricultural University, Beijing, People’s Republic of China; 2 College of Food Science and Nutritional Engineering, China Agricultural University, Beijing, People’s Republic of China

**Keywords:** Anthocyanin biosynthesis, Differentially expressed gene, Female flower tissue, Fig (*Ficus carica* L,), Light deprivation, Fruit peel, RNA-Seq, Transcription factor

## Abstract

**Background:**

Color directly affects fruit quality and consumer preference. In fig syconia, the female flower tissue is contained in a receptacle. Anthocyanin pigmentation of this tissue and the peel differs temporally and spatially. A transcriptome study was carried out to elucidate key genes and transcription factors regulating differences in fig coloring.

**Results:**

Anthocyanins in the female flower tissue were identified mainly as pelargonidin-3-glucoside and cyanidin-3-rutinoside; in the peel, the major anthocyanins were cyanidin 3-O-glucoside and cyanidin-3-rutinoside. Anthocyanin content was significantly higher in the female flower tissue vs. peel before fig ripening, whereas at ripening, the anthocyanin content in the peel was 5.39 times higher than that in the female flower tissue. Light-deprivation treatment strongly inhibited peel, but not female flower tissue, anthocyanin pigmentation. RNA-Seq revealed 522 differentially expressed genes (recruited with criteria log_2_ ≥ 2 and *P* < 0.05) at fig ripening, with 50 upregulated and 472 downregulated genes in the female flower tissue. Light deprivation upregulated 1180 and downregulated 856 genes in the peel, and upregulated 909 and downregulated 817 genes in the female flower tissue. KEGG enrichment revealed significantly changed expression in the phenylpropanoid-biosynthesis and flavonoid-biosynthesis pathways in the peel, but not in the female flower tissue, with significant repression of *FcCHS*, *FcCHI*, *FcF3H*, *FcF3’H*, *FcDFR* and *FcUFGT* transcripts. Light deprivation led to differential expression of 71 and 80 transcription factor genes in the peel and female flower tissue, respectively. Yeast one-hybrid screen revealed that FcHY5 and FcMYB114 bind the promoter regions of *FcCHS* and *FcDFR*, respectively in the flavonoid-biosynthesis pathway.

**Conclusions:**

Phenylpropanoid- and flavonoid-biosynthesis pathways were differentially expressed spatially and temporally in the peel and female flower tissue of fig syconia; pathway expression in the peel was strongly regulated by light signal. Differentially expressed transcription factors were recruited as candidates to screen important expression regulators in the light-dependent and light-independent anthocyanin-synthesis pathway. Our study lays the groundwork for further elucidation of crucial players in fig pigmentation.

**Electronic supplementary material:**

The online version of this article (10.1186/s12870-019-1816-9) contains supplementary material, which is available to authorized users.

## Background

Anthocyanins are water-soluble secondary metabolites of the flavonoid metabolic pathway, which accumulate in the cell vacuole mainly in the form of glycosides during plant tissue pigmentation. The type and amount of anthocyanin accumulation is determined by genetic background and affected by environmental factors [1]. The biosynthetic pathways of anthocyanins in both monocotyledons and dicotyledons have been well studied [2, 3]. The structural genes in the anthocyanin-biosynthesis pathway, such as chalcone synthase (*CHS*), chalcone isomerase (*CHI*), flavanone 3-hydroxylase (*F3H*), flavanone 3′-hydroxylase (*F3’H*), dihydroflavonol-4-reductase (*DFR*), anthocyanin synthase (*ANS*) and UDP-glucose:flavonoid-3-O-glucosyltransferase (*UFGT*), and the important transcription factors, have been cloned and functionally validated [4].

Members of three transcription factor families—MYB, bHLH and WD40—play pivotal roles in the regulation of anthocyanin- and other flavonoid-biosynthesis pathways by recruiting and forming different MYB–bHLH–WDR complexes [5]; MYBs play a leading role. In *Arabidopsis* and grapevine, bHLH-partner-independent MYBs were revealed in flavonoid biosynthesis [6]. MdMYB10 was found to be the key regulator in apple peel red color development, while anthocyanin synthesis in the red flesh of apple was induced by MdMYB110a [7]. In grapevine, VvMYBA1 and VvMYBA2 were revealed as key regulators in anthocyanin biosynthesis [8], whereas in pear, the key MYB was identified as PcMYB10 [9].

Fig (*Ficus carica* L.) is one of the world’s earliest domesticated fruit trees [10]. Today, it is a commercially grown cash crop in Mediterranean countries, the USA, China, Japan and southern hemisphere countries. The fig fruit has long been regarded as a valuable source for its attractive taste, antioxidant properties and rich supply of nutritive minerals. The fruit (syconium) can be consumed fresh, dried or processed. The process of syconium development presents a typical double-sigmoid curve including two rapid growth phases (phases I and III) separated by a slow growth phase (phase II) [11]. The color of the fig peel is determined by the relative concentrations of pigments such as anthocyanins, chlorophylls and carotenoids [12]. Anthocyanin formation and chlorophyll degradation in the peel occur mainly in the fruit’s second rapid growth period, in parallel to fruit ripening, sugar accumulation and formation of other important quality traits [13]. Fig flesh develops from the female flower tissue, and can be pigmented or non-pigmented, depending on the cultivar. The main anthocyanins in fig fruit are cyanidin-3-rutinoside, cyanidin-3-glucoside and pelargonidin derivatives [14]. Cyanidin-3,5-O-diglucoside and cyanidin-O-malonyl-hexoside have also been identified in fig peel [15]. Unlike apple, orange, peach, pear and other fruit whose major cultivars usually have anthocyanin-free flesh, in fig, red-flesh cultivars such as Brown Turkey, Qingpi and others are grown worldwide or regionally as main fresh fig selections.

Fruit coloration can be strongly and differentially affected by light. Insufficient light causes poor fruit coloration in apples, pears, and grapes [16, 17], while improved light exposure enhances anthocyanin pigmentation, especially in the fruit peel [18, 19]. A model of light-induced or darkness-inhibited anthocyanin biosynthesis has been well established in plants. In the dark, the bZIP transcription factor ELONGATED HYPOCOTYL5 (HY5) and the anthocyanin biosynthesis-regulating MYBs are ubiquitinated and degraded via the 26S proteasome pathway; with light exposure, on the other hand, light-activated photoreceptors inhibit the activity of COP1 ubiquitin E3 ligase, and stabilization of HY5 and MYB proteins activates anthocyanin biosynthesis [20]. Fig peel coloration is repressed by shading, and inhibition of *FcANS1* expression was revealed [21]. Nevertheless, the key MYB(s) in fig anthocyanin pigmentation is unknown, and it is not clear whether light deprivation affects the expression of genes upstream of *ANS* in the anthocyanin-biosynthesis pathway or the underlying molecular mechanisms.

In this study, types and contents of anthocyanins in fig peel and female flower tissue were monitored at four sampling points during ‘Zibao’ fig fruit development. In addition, a light-deprivation treatment was performed in developmental phase I. The results revealed that the specific anthocyanins, their accumulation pattern and their response to light deprivation differ in the peel vs. female flower tissue. RNA-Seq demonstrated that light deprivation induces large-scale changes in gene expression in both the peel and female flower tissues, but the phenylpropanoid- and flavonoid-synthesis pathways were only significantly repressed in the peel. The interactions between FcMYB114 and the promoters of key structural genes in the anthocyanin-biosynthesis pathway were screened by yeast one-hybrid test. Our study provides new information on fig anthocyanin pigmentation, the structural genes, and possible regulators involved in light-dependent and light-independent coloring.

## Methods

### Plant, material and treatment

Common fig cv. Zibao is cultivated at the Shangzhuang Experimental Station of China Agricultural University (40°23′N, 116°49′W), Haidian District, Beijing. Trees were 5 years old and planted 2 m × 3 m in a greenhouse. Fruit in the middle stage of phase I, in the middle and late stages of phase II and in the late stage of phase III were sampled and termed T1, T2, T3 and T4, respectively. There were three biological replicates per sample, each with 20 fruit collected randomly from 10 trees. From each sample, 20 fruit were used for physiological data analysis, and the other 40 fruit were used for sampling the peel (about 2 mm thick) and female flower tissue (about 10 g weight), which were carefully excised with a scalpel. The peel and female flower tissue were immediately frozen in liquid nitrogen and stored at − 80 °C for further use.

Double-layer opaque paper bags, black inside and light brown outside (150 mm × 180 mm, Zhengguo Paper Bag, Zhengzhou Fruit Research Institute, Chinese Academy of Agricultural Sciences), were used for the light-deprivation treatment. For light-deprivation and control treatments, 60 fruit in the middle stage of phase I were randomly selected, 30 fruit were deprived of light by bagging and the other 30 were used as the control under natural light; 10 fruit were classified as one treatment group, and triplet biological replicates were set for both the light-deprived and control figs. All fruit were collected at the end of phase III; female flower tissue and peel were carefully excised with a scalpel, immediately frozen in liquid nitrogen in the field, brought back to the laboratory and stored at − 80 °C for further analysis. The peel and female flower tissue from the light-deprivation treatment were termed LD-P and LD-F respectively; those of the control group were labeled T4-P and T4-F, correspondingly.

### Fruit quality

The transverse and longitudinal diameters of the fruit were measured with a Vernier caliper, and the fruit shape index was the ratio of the longitudinal to transverse diameters. Fruit texture was measured using a durometer (Fujiwara FHM-1, Japan). Fruit total soluble solids content was measured with a hand-held refractometer (ATAGO PAL-1, Japan). Titratable acid content was determined by NaOH titration. Excel 2016 was used for data sorting and Origin 8.5 for chart drawing. Correlation analysis was performed using SPSS 19.0 (SPSS Inc., Chicago, IL, USA). Data from all analyses were expressed as average and standard error. The threshold of significance was set at *P* < 0.05.

### Anthocyanin extraction and identification

The samples for anthocyanin determination were finely ground in liquid nitrogen. About 1 g of powder was added to 5 mL of 1% HCl–methanol, leached overnight at 4 °C in the dark and then centrifuged; the sediment was washed twice with 5 mL of 1% HCl–methanol, and the supernatants were combined. The supernatant volume was adjusted to 20 mL, filtered through a microporous membrane (diameter 13 mm, pore size 0.22 μm, Advantec, CA, USA). Anthocyanins were separated in an HPLC system (Agilent 1220, Waldbronn, Germany). A Waters Symmetry C18 column (5 μm, 4.6 mm × 150 mm) was used; the loading volume was 20 μL, mobile phase A was 10% formic acid, mobile phase B was acetonitrile. The linear gradient elution design was: 0–13 min – acetonitrile 0–20%, 20 min – acetonitrile 40%, 25 min – acetonitrile 0%; column temperature was 25 °C, flow rate was 1 mL/min, and detection wavelength was 520 nm. A standard curve was prepared using cyanidin 3-O-galactoside (Beijing Solarbio Science & Technology Co. Ltd., Beijing, China).

### RNA-Seq and annotation

Total RNA was extracted from the fig materials by the CTAB method [22]. RNA concentration and purity were measured in a NanoDrop 2000 spectrophotometer (NanoDrop Technologies, Wilmington, DE, USA). RNA integrity was determined by 1% agarose gel electrophoresis, and RNA concentration was normalized (RIN ≥ 7), mRNA was isolated from 2 μg total RNA using oligo-dT magnetic beads; cDNA was synthesized using a cDNA Synthesis Kit (TaKaRa, Japan) and linking the sequencing adapter to both ends; the library preparations were sequenced on an Illumina HiSeq 4000 platform. The unigene sequence was compared to the previously completed transcriptome database (RSEM software) using HiSat2 (http://ccb.jhu.edu/software/hisat2/index.shtml) sequencing-alignment software [23], and the improved BWT algorithm was used to efficiently compare the sequencing reads to the reference database using Bowtie 2 [24]. The whole set of annotated genes was submitted to the National Center for Biotechnology Information (NCBI) SRA database (accession number PRJNA494945).

### Gene-expression analysis

Gene-expression level was expressed as fragments per kilobase of exon model per million mapped reads (FPKM). EdgeR software (http://www.bioconductor.org/packages/2.12/bioc/html/edgeR.html) was used for analysis of differentially expressed genes (DEGs) [25]. DEGs were recruited by |log2FC| ≥ 1 and *P*-value < 0.05. Enrichment analyses were performed using the software GOatools (https://github.com/tanghaibao/GOatools) and Fisher’s exact test with *P* < 0.05 [26]. KEGG pathway-enrichment analysis was performed using KOBAS software (http://kobas.cbi.pku.edu.cn/home.do) with a corrected *P*-value < 0.05 [27].

### Anthocyanin-biosynthesis pathway gene isolation and sequence alignment

#### Gene cloning

PCR primers for isolation of *CHS*, *CHI*, *F3H*, *F3’H*, *DFR* and *UFGT* were designed based on the six complete fig structural gene sequences predicted by our RNA-Seq database (Additional file 1: Table S1). The full-length gene sequences were cloned from the fig ‘Zibao’ T4 peel cDNA library. PCR was performed in a 20-μL reaction system containing 1 μL first-strand cDNA, 1 μL each of 10 μM forward and reverse primers, 7 μL DEPC-treated water, and 10 μL of 2× Taq PCR MasterMix (Tsingke, Beijing, China). The PCR conditions were as follows: initial denaturation at 94 °C for 5 min followed by 30 cycles of denaturation at 94 °C for 30 s, annealing at 56 °C for 30 s, extension at 72 °C for 1.5 min, and a final extension at 72 °C for 10 min. The PCR products were analyzed by electrophoresis on 1.0% agarose gels, and purified using an agarose gel purification extraction kit (Axygen, Corning, NY, USA), ligated into the pMD19-T vector (TaKaRa, Dalian, China), transformed into *E. coli* DH5α cells, and positive clones were selected for sequencing (Tsingke Biological Technology Co. Ltd., Beijing, China). The structural gene sequences were analyzed by protein family searches using BLAST in NCBI (http://www.ncbi.nlm.nih.gov/). Sequence alignment was performed using ClustalX software version 1.8331 (http://bips.u-strasbg.fr/fr/Documentation/ClustalX/#G).

#### qRT-PCR verification

RNA extraction and quality check were the same as for the RNA-Seq. Reverse transcription was performed using HiFi-MMLV cDNA First-Strand Synthesis Kit (Invitrogen, Carlsbad, CA, USA). Based on the transcriptome data of T1, T2, T3, T4 and light-deprived fig peels and female flowers, the expression level of 19 color-related genes was validated. The primers used for qRT-PCR are listed in Additional file 1: Table S2. The PCR was performed with an ABI 7500 Fast Real-Time Detection System (Applied Biosystems, Waltham, MA, USA) using the Ultra SYBR Mix Kit (TaKaRa, Dalian, China). The amplification system consisted of 10 μL Ultra SYBR Premix System II, 0.5 μL of 10 μM upstream primer, 0.5 μL of 10 μM downstream primer, 2 μL template, and double-distilled water to a total volume of 20 μL. The amplification program was 95 °C for 10 min, followed by 40 cycles of 95 °C for 5 s and 60 °C for 32 s. Relative quantitative analysis of gene expression was performed by the 2^-ΔΔCT^ method using β-actin as the reference gene. Three technical replicates were carried out for each sample to ensure reproducibility and reliability. Statistical analysis of variance (ANOVA) followed by Duncan’s new multiple-range test was performed with SPSS version 19.0. The significance level was set to *P* < 0.05.

#### Promoter analysis and yeast one-hybrid assay

cDNA sequences of *FcCHS* (c33458_g3) and *FcDFR* (c46884_g6) from the present study were blasted against the published fig genome [28]. Primers for cloning of the two genes’ promoters were designed according to the 5′-upstream sequences by Primer 5.0 with CHS-f (AGGGCACATCTCCAAAACTTTTC) and CHS-r (TGCGCCTTTCGGATTTCGTATAC), DFR-f (TTGTCACCCTTCCATGTCAATC) and DFR-r (GTCACACAGACAGTTTCACC). Genomic DNA was isolated from the mixed sample of peel and female flower tissue of ‘Zibao’ fig using the CTAB method [29]. PCR was carried out with a standard 20-μL reaction system using Q5® High-Fidelity DNA Polymerase (New England Biolabs, Ipswich, MA, USA). The amplification products were sequenced and *cis-*acting regulatory elements were predicted by PlantCARE (http://bioinformatics.psb.ugent.be/webtools/plantcare/html/) and PLACE (http://www.dna.affrc.go.jp/PLACE/) databases.

A yeast one-hybrid system (Y1H Gold) was used to screen the relationship between FcHY5 protein and the *FcCHS* promoter, and FcMYB114 protein and the *FcDFR* promoter. As the effector construct, the open reading frames (ORFs) of *FcHY5* and *FcMYB114* were cloned into the SmaI and SacI sites of the pGAD-T7 vector. The *FcCHS* and *FcDFR* promoter sequences were inserted upstream of the AbA^r^ reporter gene in the pABAi vector. The bait reporter strain was created by homologous recombination into the genome of Y1H Gold, resulting in the following yeast strains: AD-empty/pABAi-pFcCHS, AD-*FcHY5*/pABAi-pFcCHS; AD-empty/pABAi-pFcDFR, *FcMYB114*/pABAi-pFcDFR and AD-p53/pp53. The yeast cells were selected on synthetic drop-out media lacking leucine with AbA, and positive colonies were spotted onto glucose plates (2%) and incubated at 28 °C for 3 days [30].

## Results

### Fig fruit pigmentation

**Fig. 1:**
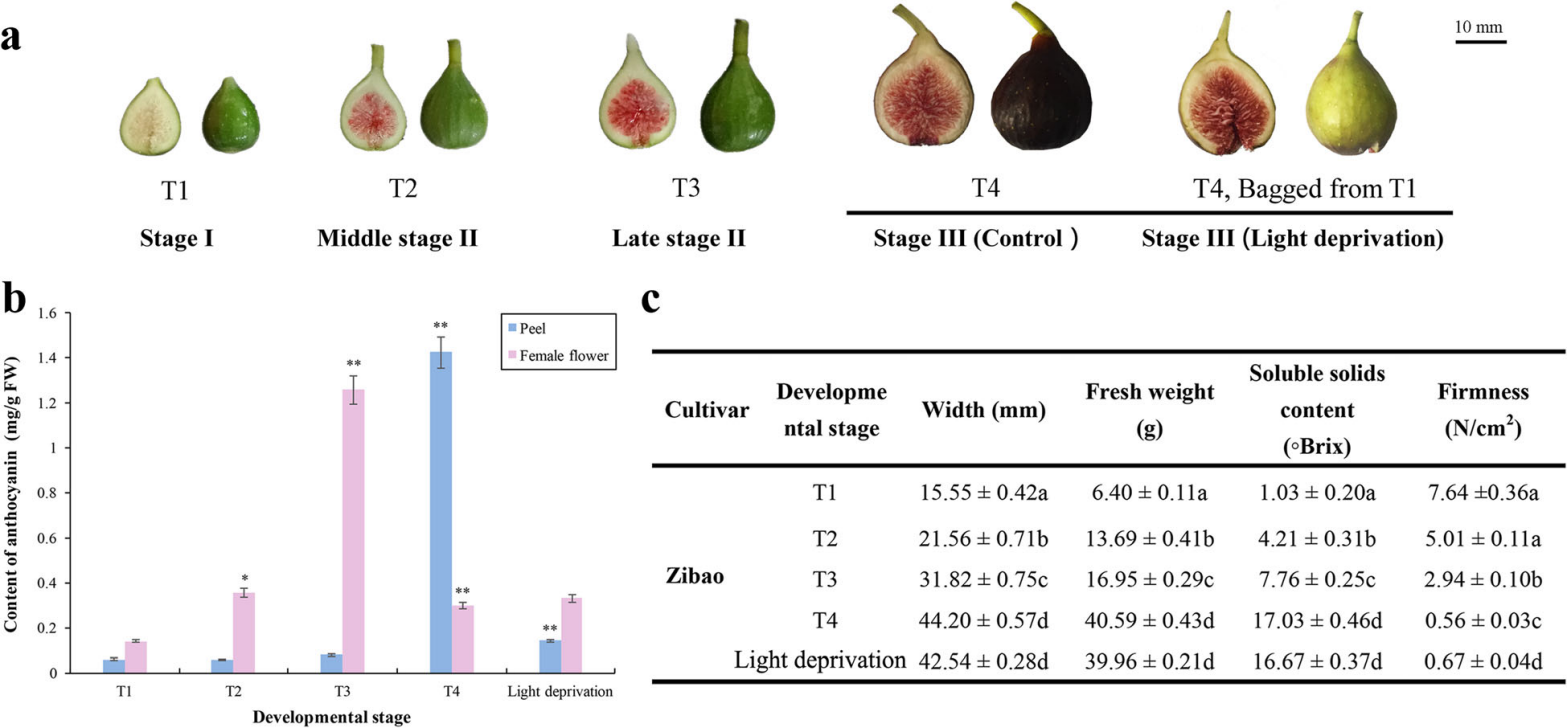
The phenotype of fig (*Ficus carica* L.) cv. Zibao at young and mature phases. **a** Developmental stages T1, T2, T3, T4 and light-deprivation treatment. **b** Fig fruit quality. **c** Anthocyanin content in peel and female flower tissue

The pigmentation of fig peel and female flower tissue showed obvious spatial and temporal patterns. Anthocyanins began accumulating in the female flower tissue after the fruit entered phase II, demonstrated a slow increase during the long period from T2 to T3, peaked at T3 with 1.256 mg/g, then decreased at T4 (Fig. 1a, b). Anthocyanin accumulation in the peel occurred very rapidly in phase III, with peak content in T4 of 1.404 mg/g, 5.39 times that of the corresponding female flower tissue sample (Fig. 1a, b). Furthermore, monitoring of fig syconium development revealed T4 as the ripening phase with rapidly increasing syconium size and weight, strongly increasing total soluble solids content and dramatically decreasing fruit firmness (Fig. 1c). Anthocyanin pigmentation in the peel paralleled fig ripening, whereas coloration of the female flower tissue did not.

Under light deprivation, the fig peel was yellowish with a slightly green hue (Fig. 1a, Light deprivation); anthocyanin content in the peel was repressed 11.8-fold compared to the control (Fig. 1b). The female flower tissue pigmentation did not seem to be affected by light deprivation (Fig. 1a, Light deprivation), and no significant difference in anthocyanin content was detected compared to controls. With respect to fruit quality parameters, except for significantly higher fruit firmness in the light-deprived vs. control fruit, no other indicators were found to differ significantly (Fig. 1c).

**Fig. 2:**
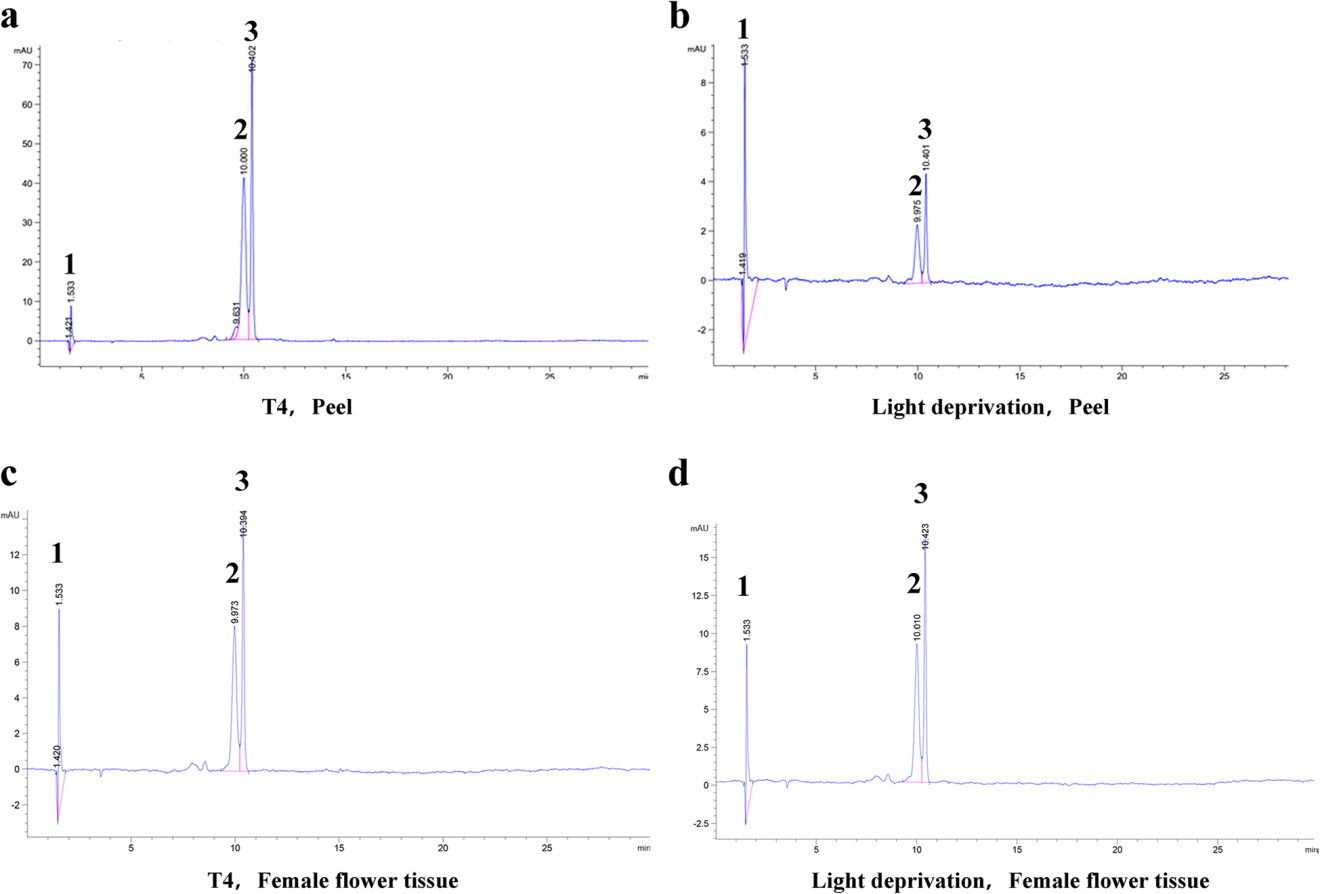
Anthocyanins in fig peel and female flower tissue. **a** Anthocyanins in the peel at stage T4. **b** Anthocyanins in the peel after light-deprivation treatment. **c** Anthocyanins in the female flower tissue at stage T4. **d** Anthocyanins in the female flower tissue after light-deprivation treatment

The types of anthocyanin also differed in fig peel vs. female flower tissue. Three anthocyanins were determined by HPLC following the sequence of peak emergence: pelargonidin-3-glucoside (1), cyanidin 3-O-glucoside (2) and cyanidin-3-rutinoside (3) (Fig. 2). Cyanidin 3-O-glucoside and cyanidin-3-rutinoside were the two main anthocyanins in the fig peel, and their contents decreased under light deprivation (Fig. 2a, b). In the female flower tissue, pelargonidin-3-glucoside and cyanidin-3-rutinoside were the two main anthocyanins, with no significant difference in content after light deprivation (Fig. 2c, d). It is interesting to note that pelargonidin-3-glucoside was consistently expressed in the peel and female flower with no influence of light intensity.

### Transcriptomic analysis

**Fig. 3:**
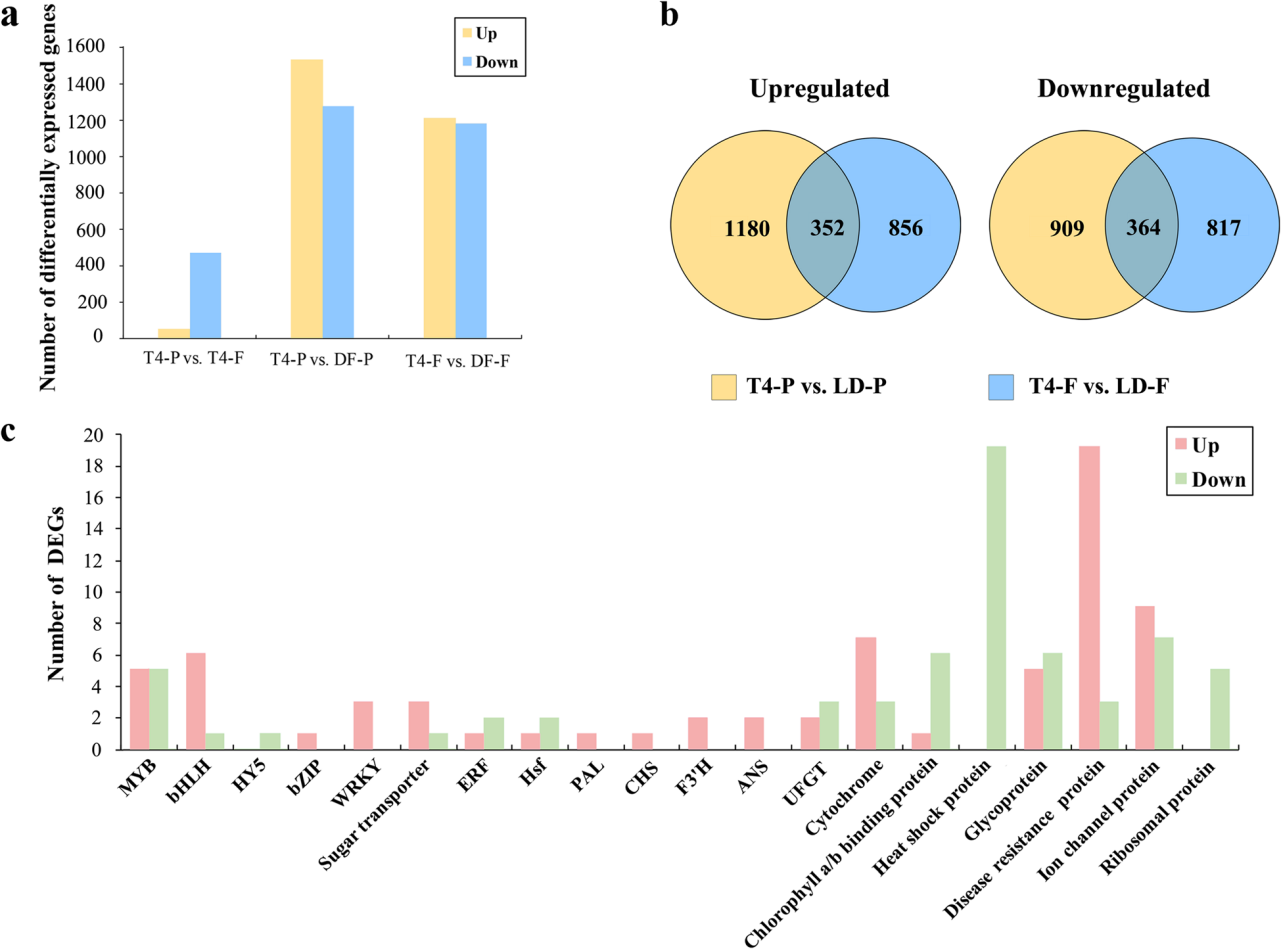
Differentially expressed genes (DEGs) between fig peel and female flower tissue and induced by light deprivation. **a** DEGs of the three comparison groups. DEGs were recruited by *P* < 0.05 and |log_2_FC| ≥ 1. **b** Venn diagram showing shared and unique DEGs in the peel and female flower tissue after light-deprivation treatment. **c** Category distribution of shared DEGs in peel and female flower tissue after light-deprivation treatment. T4-P, peel at stage T4; T4-F, female flower tissue at stage T4; LD-P, stage 4 peel following light-deprivation treatment; LD-F, stage 4 female flower tissue following light-deprivation treatment

Peel and female flower tissue of T4 phase control and light-deprived figs were analyzed by RNA-Seq and bioinformatics, each sample in 3 biological replicates. The four respective cDNA libraries yielded 8.40, 7.39, 9.57 and 9.58 Gb raw reads (Additional file 1: Table S3). After deleting the low-quality reads and removing the linker sequences, 7.88, 6.75, 9.12 and 9.14 Gb of clean data from the phase III control peel and female flower tissue (T4-P and T4-F, respectively), and light-deprived peel and female flower tissue (LD-P and LD-F, respectively) libraries were obtained, respectively. The mapping ratios to the reference database were 91.48, 91.37, 89.85, and 91.32%, respectively (Additional file 1: Table S3). With criteria *P* < 0.05 and |log2FC| ≥ 1, 522 DEGs were found in “T4-P vs. T4-F” with 50 upregulations and 472 downregulations. Light deprivation resulted in 2805 DEGs in the peel (T4-P vs. LD-P group) and 2389 DEGs in the female flower tissue (T4-F vs. LD-F group), with 1532 upregulated and 1273 downregulated in the peel, and 1208 upregulated and 1181 downregulated in the female flower tissue (Fig. 3a).

Fig peel and female flower tissue shared 716 DEGs under light deprivation, of which 352 were upregulated and 364 were downregulated (Fig. 3b). The list and Gene Ontology (GO) term assignment of all shared DEGs are shown in Additional file 1: Table S4. The shared DEGs were further screened by criteria |log2FC| ≥2 and FRKM ≥10; the anthocyanin synthesis-related DEGs were mainly classified into five transcription factor families: MYB (5 upregulated, 5 downregulated), bHLH (6 upregulated, 1 downregulated), WRKY (3 upregulated), ERF (1 upregulated, 2 downregulated) and HY5 (1 downregulated), and anthocyanin-biosynthesis pathway structural genes: *PAL* (1 upregulated), *CHS* (1 upregulated), *F3’H* (2 upregulated), *FLS* (1 upregulated), *ANS* (2 upregulated) and *UFGT* (1 upregulated, 2 downregulated) (Fig. 3c**,** Additional file 1: Table S4).

Bioinformatic analysis assigned DEGs of “T4-P vs. T4-F”, “T4-P vs. LD-P” and “T4-F vs. LD-F” to GO categories Biological Process, Cellular Component and Molecular Function, respectively (Additional file 1: Figure S1). KEGG enrichment revealed flavonoid biosynthesis, phenylpropanoid biosynthesis, and ubiquinone and other terpenoid-quinone biosynthesis as significantly differently expressed pathways (*P* < 0.01) between the peel and female flower tissues at the fig ripening phase (T4-P vs. T4-F). Light deprivation induced significant expression changes in flavonoid biosynthesis, phenylpropanoid biosynthesis, and protein processing in endoplasmic reticulum pathways in the fig peel (T4-P vs. LD-P), and correspondingly, protein processing in the endoplasmic reticulum and plant hormone signal transduction were enriched in the female flower tissue (T4-F vs. LD-F) (Table 1).

**Table 1:** Significant KEGG pathways (corrected *P*-value ≤0.01) of differentially expressed genes (DEGs) in ‘Zibao’ peel and female flower tissues following light deprivation

Pathway	DEGs with pathway annotation^a^	All genes with pathway annotation	*P*-value	Corrected *P*-value	Pathway ID
T4-P vs. T4-F					
1 Flavonoid biosynthesis	18	52	3.39E-20	4.34E-18	ko00941
2 Phenylpropanoid biosynthesis	16	182	5.70E-10	3.65E-08	ko00940
3 Ubiquinone and other terpenoid-quinone biosynthesis	9	78	4.39E-07	1.87E-05	ko00130
4 Drug metabolism - cytochrome P450	7	89	8.44E-05	0.002306851	ko00982
5 Phenylalanine metabolism	7	90	9.01E-05	0.002306851	ko00360
6 Circadian rhythm - plant	5	41	0.000136246	0.00249136	ko04712
7 Stilbenoid, diarylheptanoid and gingerol biosynthesis	4	28	0.000379383	0.006070124	ko00945
8 Metabolism of xenobiotics by cytochrome P450	6	88	0.000557625	0.007930668	ko00980
T4-P vs. LD-P					
1 Flavonoid biosynthesis	22	52	2.61E-11	6.96E-09	ko00941
2 Phenylpropanoid biosynthesis	28	182	9.31E-06	0.001243105	ko00940
3 Protein processing in endoplasmic reticulum	38	318	5.05E-05	0.002694049	ko04141
4 Plant hormone signal transduction	29	227	0.000135188	0.006015865	ko04075
T4-F vs. LD-F					
1 Protein processing in endoplasmic reticulum	36	318	4.70E-06	0.000872223	ko04141
2 Plant hormone signal transduction	28	227	1.28E-05	0.000872223	ko04075

*T4-P* peel at stage T4, *T4-F* female flower tissue at stage T4, *LD-P* stage 4 peel following light-deprivation treatment, *LD-F* stage 4 female flower tissue following light-deprivation treatment

^a^ FDR < 0.05 and absolute value of log_2_ ratio ≥ 2 (2-fold) as the threshold

### DEGs in the flavonoid biosynthesis pathway

**Fig. 4:**
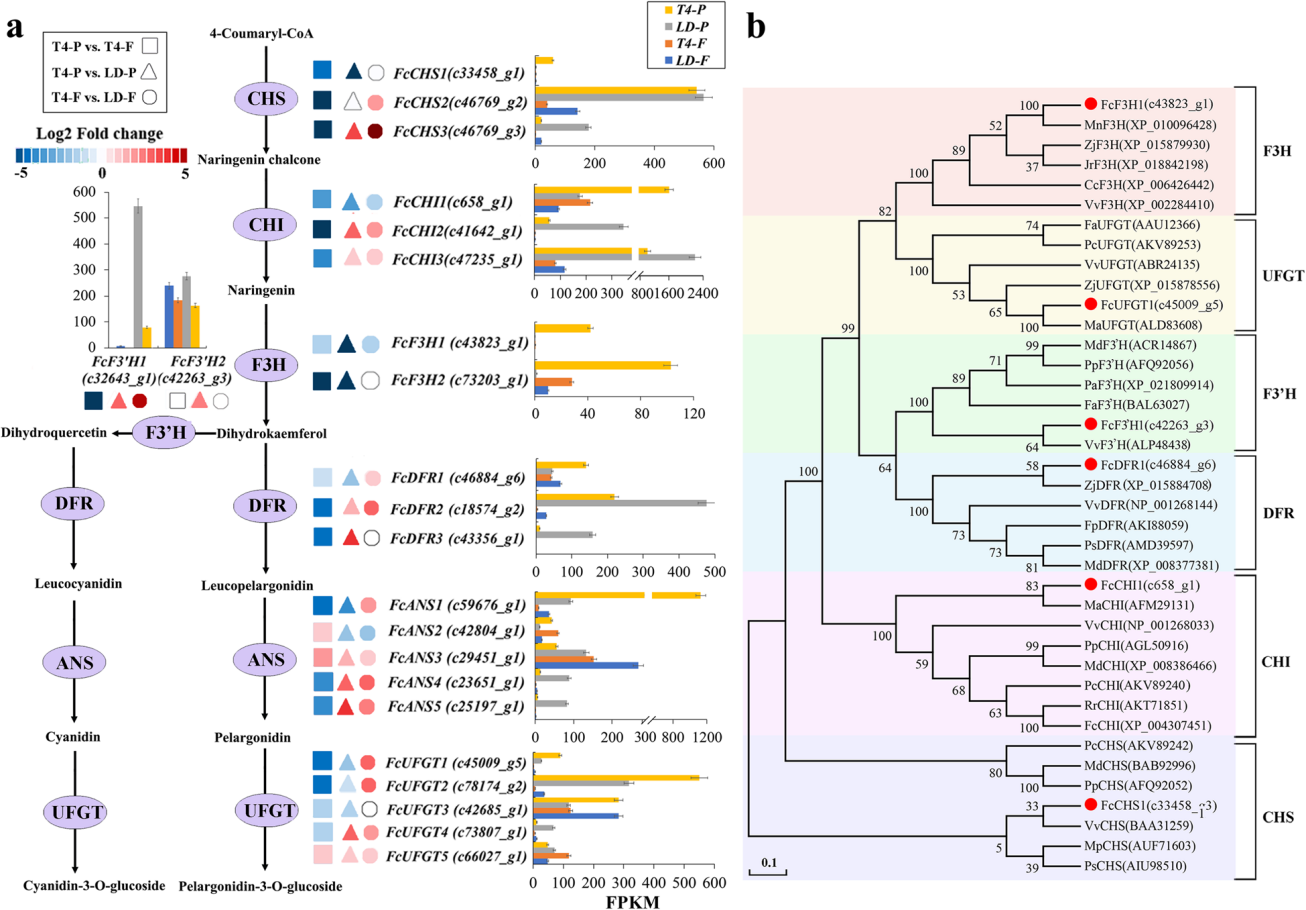
Differentially expressed structural genes (DEGs) of anthocyanin-biosynthesis pathway induced by light deprivation. **a** DEGs were recruited by |log_2_FC| ≥ 2 or FRKM ≥50. Blue and red boxes indicate downregulated and upregulated transcripts, respectively. Expression patterns are indicated at the side of each gene. CHS: chalcone synthase; CHI: chalcone isomerase; F3H: flavanone 3-hydroxylase; F3’H: flavanoid 3′-hydroxylase; DFR: dihydroflavonol 4-reductase; ANS: anthocyanidin synthase; UFGT: UDP glucose-flavonoid 3-O-glcosyl-transferase. **b** Phylogenetic clustering with corresponding genes of other fruit species. T4-P, peel at stage T4; T4-F, female flower tissue at stage T4; LD-P, stage 4 peel following light-deprivation treatment; LD-F, stage 4 female flower tissue following light-deprivation treatment

Seven structural genes—*CHS*, *CHI*, *F3H*, *F3 ´H*, *DFR*, *ANS*, *UFGT*—sequentially catalyze anthocyanin biosynthesis. Potentially important differentially expressed structural genes in the fig fruit response to light deprivation were screened by |log_2_FC| ≥ 2 or at least one FRKM ≥50. Three *CHS* genes were recruited. At fig ripening, their transcripts were downregulated 6.64-, 7.11-, 9.7-fold in the female flower tissue compared to the peel (Fig. 4a). In the peel, *FcCHS1* was downregulated 7-fold by light deprivation, and the other two *CHS* genes were significantly upregulated. In “T4-F vs. LD-F”, *FcCHS2* and *FcCHS3* were significantly upregulated.

Among the three recruited *CHI* genes, *FcCHI2* was downregulated 7.28-fold in the female flower tissue vs. peel. Light deprivation led to divergent expression of *FcCHI1* and *FcCHI2* in the peel, whereas no significant change in *CHI* expression was found in the female flower tissue. Three *F3H* genes were identified: *FcF3H1* was downregulated 7.72-fold in the female flower tissue vs. peel. After light deprivation, 8.24-, 6.28-, and 2.51-fold repression was found for *FcF3H1*, *FcF3H2* and *FcF3H3*, respectively, in the peel, and a similar trend was revealed in the female flower tissue.

We screened two *F3’H* genes. The transcripts of *FcF3’H1* (c32643_g1) and *FcF3’H2* (c42263_g3,) were 8.49- and 7.52-fold lower in the female flower tissue vs. peel. Light deprivation led to 2.77- and 2.27-fold upregulation of *FcF3’H1*, and 5.09- and 3.75-fold upregulation of *FcF3’H2* in peel and female flower tissue, respectively. The transcripts of three *DFR* genes were markedly lower in the female flower tissue than in the peel; *FcDFR2* and *FcDFR3* were upregulated 1.11- and 3.92-fold in the peel by light deprivation, whereas *FcDFR1* was downregulated.

Five *ANS* genes were recruited. *FcANS1* and *FcANS5* demonstrated 6.97- and 5.17-fold higher expression in the peel than in the female flower tissue, respectively. Light deprivation significantly repressed *FcANS1* (3.61-fold), but in female flowers, it was upregulated 1.99-fold. *FcANS2* was downregulated in both peel and female flowers (1.86- and 1.74-fold) under light deprivation. Five *UFGT* genes were selected. *FcUFGT1* and *FcUFGT2* had 6.81- and 6.44-fold higher expression in the peel vs. female flower tissue at fig ripening; they were significantly downregulated (1.73- and 1.27-fold) in the peel by light deprivation, whereas in the female flower tissue, they were significantly upregulated (2.8- and 2.52-fold) (Fig. 4a).

The full sequences of *FcCHS1*, *FcCHI1*, *FcF3H1, FcF3’H1*, *FcDFR1* and *FcUFGT1* were cloned from the fig ‘Zibao’ T4 peel cDNA library. The ORFs of the six genes were 1173, 702, 1008, 1530, 1056 and 1374 bp, respectively. Blasting against GenBank (NCBI) showed that all anthocyanin-biosynthetic structural genes cloned in this study had high sequence homology with those of mulberry and other fruit species. Among them, *FcCHS1* was closely related to grape *VvCHS* with 89% identity. *FcCHI1* was closely related to mulberry *MaCHI*, with 81% identity. *FcF3H1* was most closely related to *Morus notabilis F3H*, with an identity of 88%. *FcF3’H1* was most closely related to *VvF3’H*, with 77% identity. *FcDFR1* was most closely related to jujube *ZjDFR*, with 82% identity, and *FcUFGT1* was closely related to mulberry *MaUFGT*, with 82% identity (Fig. 4b).

### Transcription factors

Transcription factors are key players in regulating the expression of structural genes in secondary metabolite biosynthesis. In our study, 25, 71 and 80 DEGs were identified as transcription factors in “T4-P vs. T4-F”, “T4-P vs. LD-P” and “T4-F vs. LD-F” comparisons, respectively. There were 3 upregulated transcription factors and 22 downregulated transcription factors in the peel compared to the female flower tissue at ripening. Light deprivation induced upregulation of 49 genes and downregulation of 23 genes in the peel, and upregulation of 45 genes and downregulation of 35 genes in the female flower tissue. The transcription factors were annotated as MYB, bHLH, AP2/ERF, WRKY, bZIP/HY5 and HSF (Table 2).

**Table 2:** Expression profiles of same and differentially expressed genes (DEGs) encoding transcription factors (TFs) in ‘Zibao’ peel and female flower tissues by light deprivation

Comparison group	Gene name	Number of same genes	Number of DEGs^a^	Upregulated DEGs	Downregulated DEGs	Description	Biological functions
T4-P vs. T4-F	MYB	78	6	0	6	MYB TFs	Cell development and anthocyanin pathway
	bHLH	79	4	2	2	Basic helix-loop-helix protein	Plant development and substance metabolism
	WRKY	65	6	0	6	WRKY DNA-binding protein	Defense responses and plant development
	AP2/ERF	59	2	0	2	Ethylene-responsive TF	Plant development and stress response
	HSF	21	4	1	3	Heat stress TF	Plant growth, development and stress response
	Other TFs	146	3	0	3		
	In total	448	25	3	22		
T4-P vs. LD-P	MYB	93	16	13	3	MYB TFs	Cell development and anthocyanin pathway
	bHLH	83	16	15	1	Basic helix-loop-helix protein	Plant development and substance metabolism
	WRKY	75	13	11	2	WRKY DNA-binding protein	Defense responses and plant development
	AP2/ERF	63	9	5	4	Ethylene-responsive TF	Plant development and stress response
	bZIP/HY5	12	3	2	1	Homeobox-leucine zipper protein	Photomorphogenesis and fruit ripening
	HSF	21	3	1	2	Heat stress TF	Plant growth, development and stress response
	Other TFs	141	10	2	8		
	In total	488	71	49	22		
T4-F vs. LD-F	MYB	76	13	12	1	MYB TFs	Cell development and anthocyanin pathway
	bHLH	81	17	8	9	Basic helix-loop-helix protein	Plant development and substance metabolism
	WRKY	64	12	10	2	WRKY DNA-binding protein	Defense responses and plant development
	AP2/ERF	59	6	3	3	Ethylene-responsive TF	Plant development and stress response
	bZIP/HY5	12	6	3	3	Homeobox-leucine zipper protein	Photomorphogenesis and fruit ripening
	HSF	22	6	2	4	Heat stress TF	Plant growth, development and stress response
	Other TFs	136	20	7	13		
	In total	450	80	45	35		

*T4-P* peel at stage T4, *T4-F* female flower tissue at stage T4, *LD-P* stage 4 peel following light-deprivation treatment, *LD-F* stage 4 female flower tissue following light-deprivation treatment

^a^FDR ≤ 0.05 and absolute value of log_2_ ratio ≥ 2 (2-fold) as the threshold

**Fig. 5:**
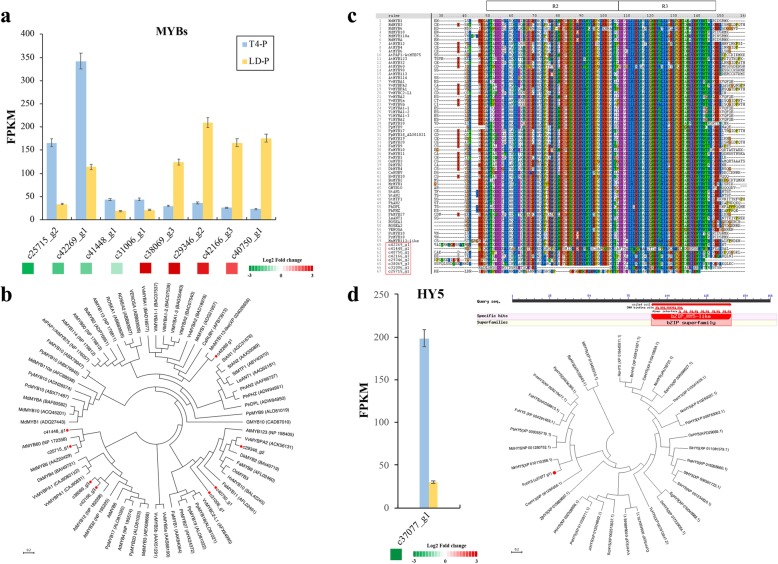
Light deprivation induces differential expression of *MYB* and *HY5* genes in fig peel. **a** Potentially important MYBs recruited by |log_2_FC| ≥ 1 and FRKM ≥20 in at least one sample. **b** Phylogenetic clustering of the recruited MYBs with anthocyanin biosynthesis-related MYBs from other plants. **c** R2R3 motif sequence alignment. **d** Expression, domain alignment and phylogenetic clustering of the fig *HY5* gene

Eight *MYB* genes were recruited from the “T4-P vs. LD-P” group by |log_2_FC| ≥1 and at least one sample FRKM ≥20. Among them, 4 genes were upregulated by light deprivation, namely c40750_g1 (2.92-fold), c42166_g3 (2.69-fold), c29346_g2 (2.52-fold) and c38069_g3 (2.07-fold); 4 genes (c25715_g2, c42269_g1, c41448_g1 and c31006_g1) were significantly downregulated by 2.28-, 1.59-, 1.23- and 1.06-fold, respectively **(**Fig. 5a**)**. A phylogenetic tree using 58 anthocyanin synthesis-related MYB protein sequences obtained from the NCBI database revealed the distribution of different clusters of the 8 fig *MYB* genes; among them, gene c42269_g1, which had the highest FPKM (342.445) of the 8, was grouped with grape MYBA1, MYBA2 and other important function-validated MYBs (Fig. 5b). Sequence analysis revealed that c42269_g1 has an R2R3 DNA-binding domain and highly variable truncated C-terminal region, which might relate to fig pigmentation regulation **(**Fig. 5c**)**. Thus, gene c42269_g1 was selected for further study.

In “T4-P vs. LD-P”, 1 *HY5* gene (c37077_g1) was downregulated 2.7-fold by light deprivation in fig peel. The gene contains a 501-bp ORF encoding a protein of 166 amino acids. The predicted secondary structure of FcHY5 showed that it has a bZIP domain at the C-terminal end from 90 to141 amino acids. Phylogenetic tree analysis showed that FcHY5 exhibits the highest homology with MnHY5 (95% identity) and MdHY5 (84% identity) from *Morus notabilis* and *Malus domestica*, respectively **(**Fig. 5d**)**.

### RT-qPCR validation

**Fig. 6:**
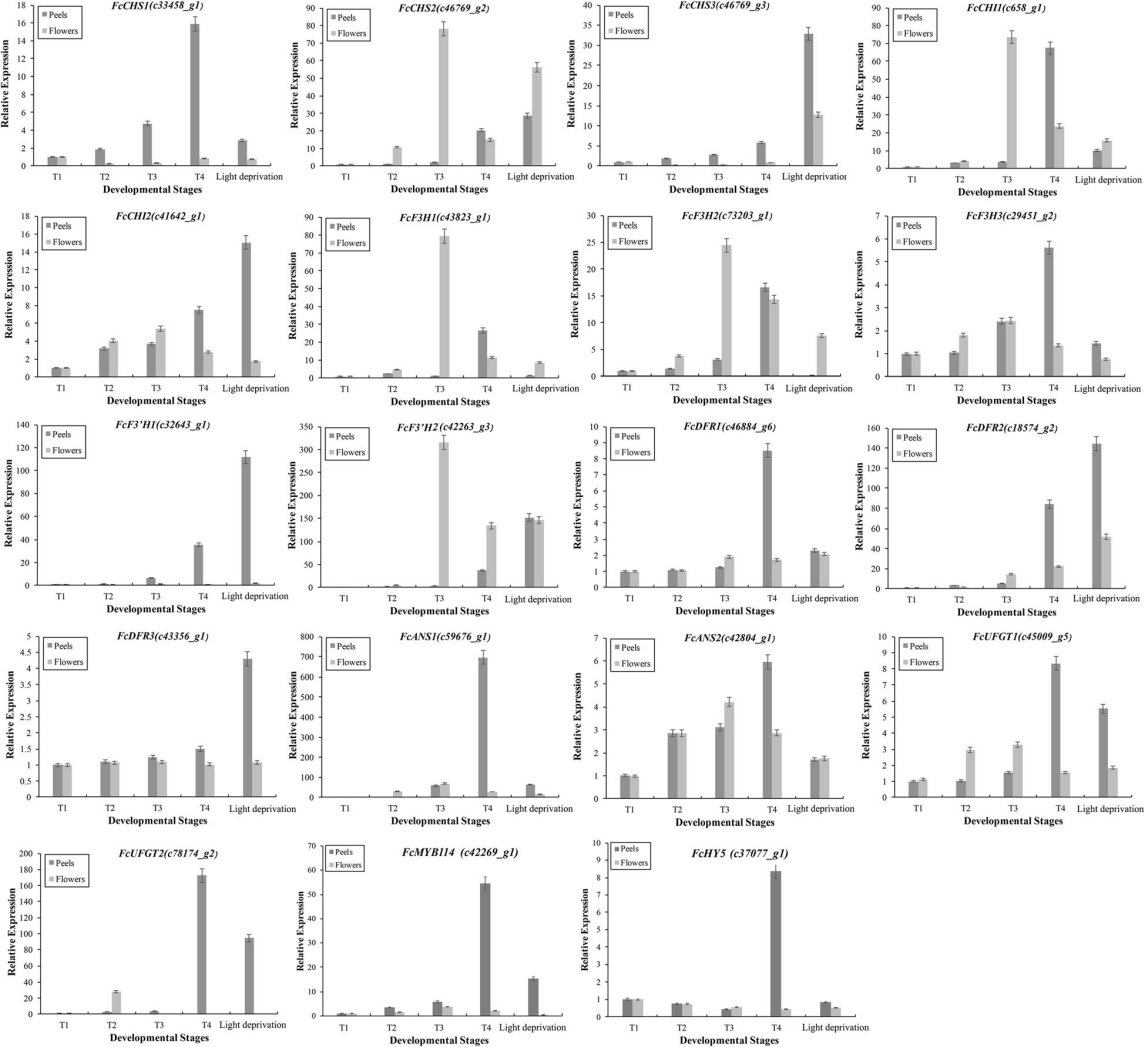
qRT-PCR validation. Nineteen unigenes from the flavonoid-biosynthesis pathway were selected to validate the RNA-Seq results. Relative expression of the genes in the peel and female flower tissue in T1, T2, T3, T4 and light-deprived samples is shown. Three biological replicates were used

To validate the key results of the RNA-Seq, we selected 19 genes from the flavonoid-biosynthesis pathway and analyzed their expression levels in T1, T2, T3, T4 and light-deprived T4 samples using qRT-PCR (Fig. 6). The expression levels of these structural genes were in line with those of the RNA-Seq results.

### Promoter sequence analysis and yeast one-hybrid validation

**Fig. 7:**
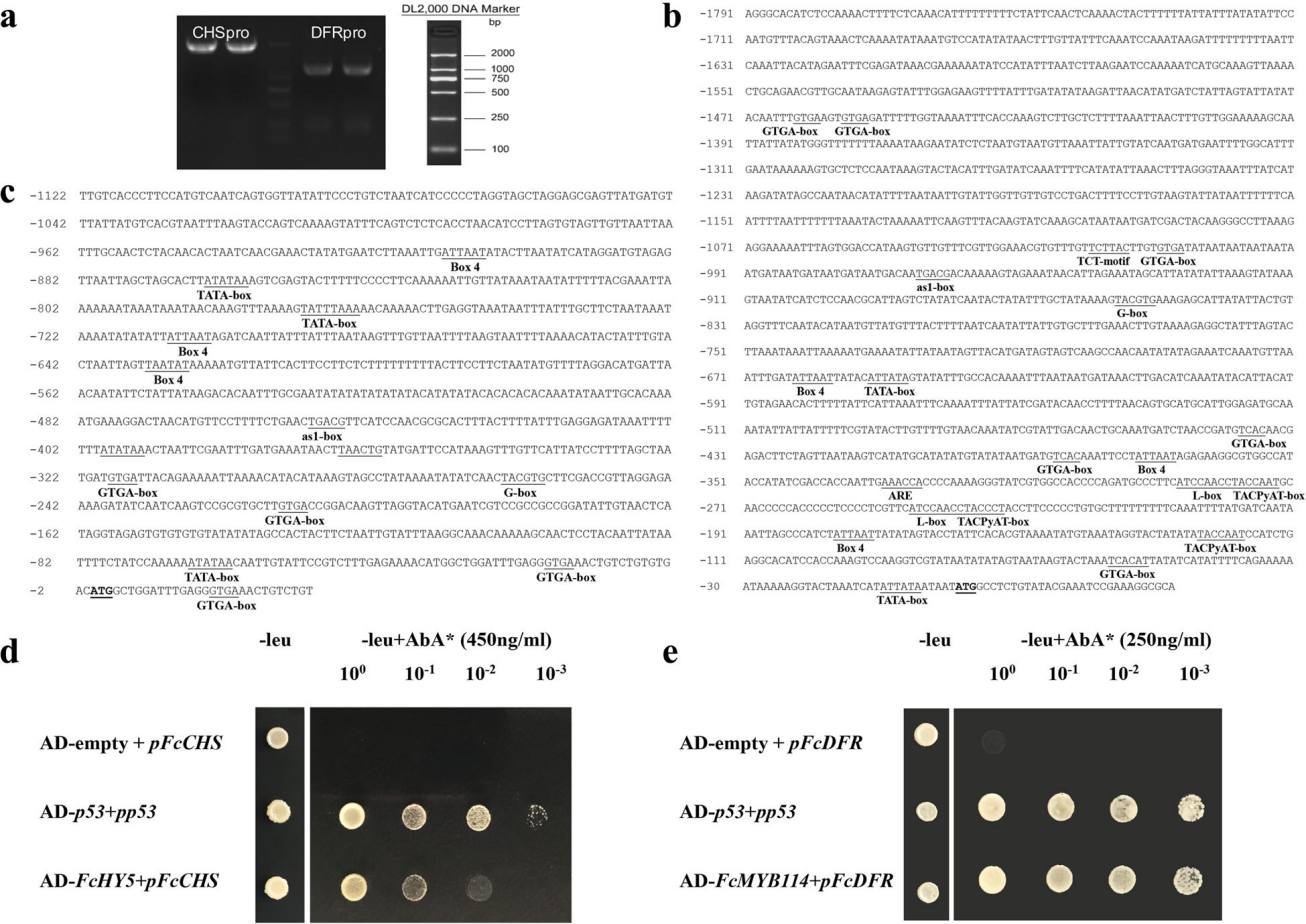
Fig *CHS* and *DFR* gene promoter clone, sequence analysis and transcription factor binding validation*.* **a** *FcCHS* and *FcDFR* gene promoter clone. **b** Nucleotide sequence of the 1791-bp upstream region of *FcCHS*. Functional elements and all putative *cis*-elements are underlined. **c** Nucleotide sequence of the 1122-bp upstream region of *FcDFR*. Functional elements and all putative *cis*-elements are underlined. **d** Yeast one-hybrid test using *FcCHS* promoter as bait and FcHY5 as prey. **e** Yeast one-hybrid test using *FcDFR* promoter as bait and FcMYB114 as prey. Representative growth status of yeast cells is shown on SD/−leucine agar media with or without AbA from triplicate independent trials. Numbers at the top of each photograph indicate relative densities of the cells

Promoter sequences of *FcCHS1* and *FcDFR1* were cloned to search for homologous *cis*-regulatory elements, with a focus on MYB-binding and light signal-responsive sites: 1791 bp and 1122 bp upstream of *FcCHS* and *FcDFR* were isolated and sequenced (Fig. 7a). In addition to the typical promoter elements, such as the TATA and CCAAT boxes, elements relating to light responsiveness, such as G-box, L-box, Box 4 and GTGA-box, were found in the *FcCHS* promoter (Fig. 7b), indicating that the transcription of *FcCHS1* could be regulated by light-induced signal-transduction elements such as HY5 [31]. The H-box, I-box and G-box elements, which are required for MYB transcription factor binding, were revealed in the *FcDFR* promoter. Note that the elements related to light responsiveness, including Box 4 and G-box, were also found in the *FcDFR1* promoter sequence (Fig. 7c).

To further validate that FcHY5 and FcMYB114 are involved in *FcCHS1* and *FcDFR1* transcription in fig, we tested FcHY5 and FcMYB114 binding to the promoters of *FcCHS1* and *FcDFR1* using the Matchmaker Y1H Gold system. Growing colonies could be seen with AD-FcHY5/pABAi-pFcCHS, *FcMYB114*/pABAi-pFcDFR and AD-p53/pp53 (positive control) strains; strains AD-empty/pABAi-pFcCHS and AD-empty/pABAi-pFcDFR served as negative controls (Fig. 7d). The results verified the effectiveness of our RNA-Seq-recruited transcription factors and their possible roles in anthocyanin biosynthesis-related structural gene transcription.

## Discussion

### Pigmentation differences between fig peel and female flower tissue

Anthocyanins are responsible for the series of colors in fig fruit at ripening. In the market, cultivars with purple peel and red flesh are most popular among consumers [15, 21]. Light deprivation significantly represses anthocyanin biosynthesis in the peel, leading to yellow–green figs, while the inside of the figs remains strawberry-colored, suggesting different regulation of fig peel and female flower tissue pigmentation.

Fig is an accessory fruit, and the divergent gene expression in its female flower tissue and surrounding receptacle has been reported for other biologically important pathways. The ripening-related gene families, including key elements and transcription factors of the ethylene signal-transduction pathway, have been shown to have differential expression patterns in the female flower tissue and receptacle, and these two parts of the fig have been suggested to have climacteric and non-climacteric characteristics, respectively; specifically, fig peel pigmentation is in line with the ethylene release peak in the fruit [32]. Color mutations are valuable materials to study the coloration regulation of figs. ‘Zibao’ is a purple peel mutation of cv. Green Peel; both the original cultivar and the mutant have red flesh at ripening, further supporting the notion that the color regulation of fig peel and female flower tissue has independent patterns [15].

The pigmentation of fig female flower tissue and peel is also affected by biological factors, such as pollination. Common fig cultivars are parthenocarpic; pollination not only leads to bigger fruit, but also markedly earlier pigmentation of the female flowers. Moreover, the color intensity of the female flower tissue seems to be stronger in the pollinated fruit than in the parthenocarpic fruit during the whole process of fig development. At harvest, the peel of pollinated fruit is darker than that of parthenocarpic fruit [33].

Fruit color mutants are usually mediated by a single gene mutation, in many cases of a key *MYB* with on–off control of anthocyanin-synthesis regulation [34, 35]. However, beyond the visible color change and correspondingly different anthocyanin contents, changes in other secondary metabolites have been revealed. Metabolomic comparison of peels of ‘Zibao’ and ‘Green Peel’ showed a significant increase in procyanidin, luteolim-3′7’-diglucoside and epicatechin in the peel of the ripening ‘Zibao’ [15]. This suggests that the same transcription factor regulating *FcANS* may also regulate other structural genes in the flavonoid-biosynthesis pathway.

### Structural genes whose expression is influenced by light deprivation

The anthocyanin-synthesis pathway has been extensively studied in model plants and economically relevant crops; genes encoding key enzymes have been cloned and their functions validated [36]. However, the key regulators controlling the different coloration of fig peel and female flower tissue are still unknown. *FcANS1* was previously cloned in our laboratory. The present study revealed that for the highest FPKM isogenes, *F3H*, *ANS* and *UFGT* had significantly higher transcripts in the peel than in the female flower tissue; *F3’H* and *DFR* transcripts were significantly higher in the female flower tissue than in the peel; *CHS* had similar transcript numbers in the two tissues, whereas the number of transcripts of two *CHIs* (*CHI1* and *CHI3*) were highest in the peel and female flower tissue, respectively, suggesting delicate mechanisms regulating the expression of the structural genes and leading to different anthocyanin-accumulation patterns in the female flower tissue and fruit peel.

Studies with red pears and grapes have shown that the key enzymes in anthocyanin synthesis are *ANS* and *UFGT* [36, 37]. In this study, we found that the high expression of *ANS* and *UFGT* was very significantly repressed by light deprivation in both the peel and female flower tissue, which is in agreement with the previous reports. Moreover, the upstream *CHS* and *CHI* expression levels were also significantly downregulated by light deprivation. Our results suggest that *CHS* and *CHI* play upstream rate-controlling roles in the anthocyanin-biosynthesis pathway, and jointly regulate the biosynthesis and accumulation of anthocyanins in fig fruit with the downstream structural genes.

### More than one MYB may regulate fig anthocyanin biosynthesis

Anthocyanins are found at various levels and with different accumulation patterns in a large number of plant organs, such as flowers, fruit peel, fruit flesh, vegetative tissues, tubers and other organs, and play important roles in facilitating reproduction and plant stress resistance. MYBs are regarded as the major determinant in anthocyanin-biosynthesis regulation [38]. They constitute one of the largest transcription factor families in plants, with more than 100 members found in *Arabidopsis* [39], kiwi [40] and other crops, which can be further assigned to more than 39 subgroups according to the specific domains and motifs. In our study, *FcMYB114* (c42269_g1) had the highest FPKM of all of the annotated *MYB* genes; with its significant downregulation by light deprivation, domain and sequence alignment and primary transactivation and binding specificity validation, it is speculated to be a major determinant *MYB* in light-induced fig peel color development.

Plants can have more than one anthocyanin-regulating MYB: in *Arabidopsis*, MYB75 (PAP1), MYB90 (PAP2), MYB113 and MYB114 have roles in anthocyanin pigmentation; in apple, peel pigmentation is controlled by MYB1 (MYBA), whereas apple flesh and foliage anthocyanin accumulation is regulated by MYB10 and MYB110a_JP [41, 42]; three MYBs—Rosea1, Rosea2 and Venosa—control different floral pigmentation patterns in *Antirrhinum majus* [43], and six anthocyanin-activating MYBs have been revealed in purple-foliaged plum (*Prunus cerasifera*) [44]. In our study, in addition to *FcMYB114*, three other R2R3 *MYBs* were recruited by their changing expression pattern and FPKM value, but further functional studies are required to confirm or exclude their role in fig anthocyanin biosynthesis.

MYB regulators of anthocyanin pigmentation are not all activators. In strawberry, FaMYB10 and FvMYB10 (from *Fragaria vesca*) activate the expression of the anthocyanin pathway, whereas FaMYB1serves as a repressor, suggested to balance the concentration of anthocyanins and other flavonoids. In grape, VvMYB4-like is a repressor of *ANS*, *DFR* and *UFGT*, which is highly expressed in the skin of berries before ripening and inhibits pigmentation [45]. In our study, four *MYB*s were found to be significantly upregulated under light deprivation. The anthocyanin-biosynthesis pathway and its regulators have been found to be highly conserved in different plants. Functional analysis of the four *MYB*s that were upregulated by light deprivation could provide us with a better understanding of the implementation of diverse fig peel and flesh color combinations.

## Conclusions

Light is one of the important external factors affecting the biosynthesis of plant anthocyanins. Low or uneven light caused by protected cultivation or canopy shading frequently leads to undesirable low coloration or non-uniform pigmentation in the peel, which decreases the fruit’s commercial and nutritional value. However, anthocyanins can accumulate in the dark or under very low light in the red-flesh fruit cultivars. An elucidation of the different regulatory mechanisms could help breeders create new cultivars with improved coloration traits for both fruit peel and flesh. In our study, anthocyanin type and contents in the fig peel and female flower tissue, and their alteration under light deprivation, were analyzed. Transcriptome analysis revealed differentially expressed pathways and specific structural anthocyanin-biosynthesis genes. A number of potential transcription factors, in particular *MYB*s, were recruited, and primary binding targets were screened. Further specific and in-depth functional studies of the *MYB*s and other recruited transcription factors could bring new information to further understand the coloration mechanism of fig fruit flesh and peel under light deprivation, and the complex regulatory mechanism of plant color formation.

## Additional file

Additional file 1: **Table S1.** Primer sequences of structural genes in the flavonoid-biosynthesis pathway used for cDNA cloning. **Table S2.** Primer sequences of flavonoid-biosynthesis pathway genes for qRT-PCR verification. **Table S3.** Summary of the sequencing assembly. **Table S4.** Shared differentially expressed genes in ‘Zibao’ peel and female flower tissue following light deprivation. **Figure S1.** GO classification of unigenes of *Ficus carica* L. syconia. The results are summarized in (a) T4-P vs. T4-F; (b) T4-F vs. LD-F; (c) T4-P vs. LD-P. GO categories: Biological Process, Cellular Component and Molecular Function. T4-P, peel at stage T4; T4-F, female flower tissue at stage T4; LD-P, stage 4 peel following light-deprivation treatment; LD-F, stage 4 female flower tissue following light-deprivation treatment. (PDF 1188 kb)

## Abbreviations

ANS: Anthocyanidin synthase
bHLH: Basic helix-loop-helix
CHI: Chalcone isomerase
CHS: Chalcone synthase
COG: Clusters of orthologous groups of proteins database
DEG: Differentially expressed gene
DFR: Dihydroflavonol 4-reductase
F3′H: Flavanone 3′-hydroxylase
F3H: Flavanone 3-hydroxylase
FPKM: Fragments per kilobase of exon model per million mapped reads
GO: Gene Ontology
KEGG: Kyoto Encyclopedia of Genes and Genomes
LAR: Leucoanthocyanidin reductase
MYB: V-myb avian myeloblastosis viral oncogene homolog
PAL: Phenylalanine ammonia-lyase
UFGT: UDP-glucose: flavonoid 3-O-glucosyltransferase
