# Supplementary material for: Differential color development and response to light deprivation of fig (Ficus carica L.) syconia peel and female flower tissues: transcriptome elucidation
Source: BMC Plant Biol. 2019 May 23;19:217. doi: 10.1186/s12870-019-1816-9 (PMC6533723; doi:10.1186/s12870-019-1816-9)
Supplement: Supplementary file 1 — Table S1. Primer sequences of structural genes in the flavonoid-biosynthesis pathway used for cDNA cloning. Table S2. Primer sequences of flavonoid-biosynthesis pathway genes for qRT-PCR verification. Table S3. Summary of the sequencing assembly. Table S4. Shared differentially expressed genes in ‘Zibao’ peel and female flower tissue following light deprivation. Figure S1. GO classification of unigenes of Ficus carica L. syconia. The results are summarized in (a) T4-P vs. T4-F; (b) T4-F vs. LD-F; (c) T4-P vs. LD-P. GO categories: Biological Process, Cellular Component and Molecular Function. T4-P, peel at stage T4; T4-F, female flower tissue at stage T4; LD-P, stage 4 peel following light-deprivation treatment; LD-F, stage 4 female flower tissue following light-deprivation treatment. (PDF 1188 kb) [file 12870_2019_1816_MOESM1_ESM.pdf]

Differential color development and response to light deprivation of fig (*Ficus carica* L.) syconia peel and female flower tissues: transcriptome elucidation

Ziran Wang[1], Miaoyu Song[1], Yunze Li[1], Shangwu Chen[2], Huiqin Ma[1*]

[1]College of Horticulture, China Agricultural University, Beijing, P.R. China

[2]College of Food Science and Nutritional Engineering, China Agricultural University, Beijing, P.R. China

Ziran Wang: wangziran@cau.edu.cn

Miaoyu Song: songmiaoyu@cau.edu.cn

Yunze Li: liyunze@cau.edu.cn

Shangwu Chen: swchen@cau.edu.cn

Huiqin Ma: hqma@cau.edu.cn

*Correspondence: Huiqin Ma, hqma@cau.edu.cn

Table S1 Primer sequences of structural genes in the flavonoid-biosynthesis pathway used for cDNA cloning

Gene name	Seq ID	Primer sequence (5'→3')	Length (bp)	Annealing temperature (°C)	
FcCHS1	c33458_g1	Forward: ATGGCCTCTGTATACGAAATCCG<br>Reverse: TTAATTAAGGGCAAGACTGCGG	1173	56	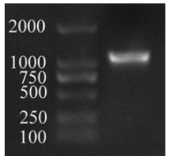
FcCHI1	c658_g1	Forward: ATGCCTCCGATGACACTTAC<br>Reverse: TCAAACTGCCAACAGTTTCT	702	58	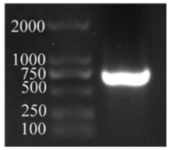
FcF3H1	c43823_g1	Forward: ATGGAGGTAGAGAGGGTCC<br>Reverse: TTATTGGGGAAGCTTGTTGAGC	1008	56	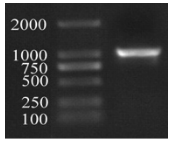
`*FcF3·H1*	c32643_g3	Forward: ATGCCTTCTGTCTCTATCATCCT<br>Reverse: TTATGCTTGATATAAGTGTTGGGCC	1530	56	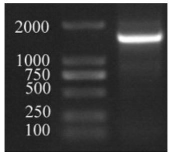
FcDFR1	c46884_g6	Forward: ATGGCAAATGCAAAACAAATAAATTTCG<br>Reverse: TTACTTAAGATCACCAAATTTCCTCGC	1056	57	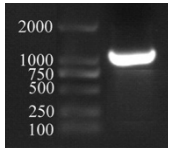

FcUFGT1	c45009_g5	Forward: ATGGCATCACCACCACCA<br>Reverse: TCACTCTTGAACTCCTCCGG	1374	58	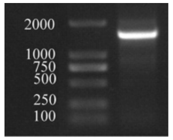

Table S2 Primer sequences of genes used for verification of digital gene-expression results by RT-qPCR

**Gene name**	**Seq ID**	**Forward primer (5'→3')**	**Reverse primer (5'→3')**
Actin	Reference gene	GCCATTCAAGCCGTGCTTT	TGGGAACAGTGTGGCTGACA
FcCHS1	c33458_g1	ACCAATCCATCTGAGGCACA	GATAATCAGCCTGCGAGACG
FcCHS2	c46769_g2	CCGTGAAGTTGGGCTTACAT	AAACCACACTTGGCTTCCAC
FcCH34	c46769_g3	CAAAGCACGTGACTCCCATC	ATTTCCTCGACGGTCACCAT
FcCHI1	c658_g1	GTAACGGGTCCGTTTGAGAA	GTTAAAGAACCGGACGGTGA
FcCHI2	c47235_g1	CACTTCCCTTCTTCCCCTTC	TGGTTGATGAAATCCCCTTC
FcF3H1	c43823_g1	GTCGGTCGGGTATTCTTTGA	TAGATGGAGGAGCCATGAGG
FcF3H2	c73203_g1	GAGGTAGAGAGGGTCCAAGC	GAAACCCCACTCTTTGCTCG
FcF3H3	c29451_g2	TGCGGGCATGGAGGATATAG	CACTTCCCCAAAAGGCCATC
FcF3·H1	c32643_g1	GTGGCGGAGCAGTTCCTTA	GAGGTGAACGGTGGTGATCT
FcF3·H2	c42263_g3	GATCCGCCACCCTAAAATCT	GGATGTGGTAGCCGTTGACT
FcDFR1	c46884_g6	CTCCTCCTCCGAATTAGCGT	CGCCTGATTCTGTTTCCTCG
FcDFR2	c18574_g2	AGGTGGAGGTAAGAGCAGCA	GATAGCCCATGCCACAACTT
FcDFR3	c43356_g1	TGTGGAGGGAAGCTTTAGTGA	GCTGCTCGCCAATAACAGTT
FcANS1	c59676_g1	CATCATGCACATTGGTGACA	GTGCTGGATATGCTGCTGAA
FcANS2	c42804_g1	GGCAGTCGTTGAAGAAGGTG	TCCCAACCGTCGACTTATCC

FcUFGT1	C45009_g5	GAGTTGATGGTGGGGCTTTC	TCACTCTTGAACTCCTCCGG
FcUFGT2	c78174_g2	CGGAGAACACGGAGAAGAAG	ATTCCCCTCCAAATTCCAAC
FcMYB114	c42269_g1	TGAGCGGGAGGAGATTGATC	GCAAACCAACAGACCCACAT
FcHY5	c37077_g1	CACTCACAAACCATCGGCAT	GCAAGGGAAAGGAAGAAGGC

Table S3 Summary of the sequencing assembly

Sequences	T4-P	T4-F	LD-P	LD-F
**Before trimming**				
Total nucleotides (bp)	8,406,965,982	7,394,743,914	9,575,327,666	9,582,970,682
Number of raw reads	55,870,328	48,971,814	63,412,766	63,463,382
Q20 percentage (%)	96.45	95.91	97.01	97.05
**After trimming**				
Total nucleotides (bp)	7,880,080,702	6,747,389,564	9,124,422,432	9,137,841,606
Number of raw reads	54,294,240	47,519,894	61,400,466	61,519,498
Q20 percentage (%)	98.20	98.02	98.42	98.43
**Mapping ratio**				
Total nucleotides of transcripts (bp)	54,294,240	47,519,894	61,400,466	61,519,498
Mapped nucleotides of transcripts (bp)	49,665,838	43,589,627	55,167,988	56,178,166
Mapped rate (%)	91.48	91.37	89.85	91.32

T4-P, peel at stage T4; T4-F, female flower tissue at stage T4; LD-P, stage 4 peel following light-deprivation treatment; LD-F, stage 4 female flower tissue following light-deprivation treatment.

Table S4 Shared differentially expressed genes in ‘Zibao’ peel and female flower tissue following light deprivation

Seq_id	Regulated	Annotation
**Transcription factor**		
c39854_g1	up	Basic helix-loop-helix protein 50
c43008_g2	up	Basic helix-loop-helix protein 75
c43099_g1	up	Basic helix-loop-helix protein 112
c66694_g1	up	Basic helix-loop-helix protein 78
c72970_g1	up	Basic helix-loop-helix protein 62
c81283_g1	up	Basic helix-loop-helix protein 62
c38338_g1	up	bZIP transcription factor 57
c41486_g1	up	Ethylene-responsive transcription factor 061
c39346_g3	up	HD-ZIP protein ATHB-15
c45256_g3	up	Heat shock factor protein HSF24
c12586_g1	up	MYB family transcription factor
c36728_g1	up	Transcription factor MYB1R1
c40543_g2	up	MYB-related protein 3R-1
c40750_g1	up	Transcription repressor MYB5
c44925_g1	up	Transcription factor MYB86
c81761_g1	up	NAC domain-containing protein 89
c23885_g1	up	WRKY transcription factor 9
c23885_g2	up	WRKY transcription factor 9
c40908_g1	up	WRKY transcription factor 72
c40266_g1	down	Basic helix-loop-helix protein 110
c29668_g2	down	Ethylene-responsive transcription factor 1
c41517_g1	down	Floral homeotic protein APETALA 2
c9228_g1	down	GATA transcription factor 2
c26517_g2	down	Heat stress transcription factor 25
c43194_g3	down	Heat stress transcription factor A-2
c37077_g1	down	Transcription factor HY5

c72503_g1	down	Transcription factor DIVARICATA
c27914_g3	down	Transcription factor DIVARICATA
c44885_g4	down	Transcription factor DIVARICATA
c41017_g1	down	Transcription factor DIVARICATA
c45044_g1	down	Transcription factor DIVARICATA
c32875_g1	down	Transcription factor NAC
c33198_g2	down	Transcription factor TCP22
c38915_g2	down	Transcription factor TCP19
**Anthocyanin-biosynthesis pathway**		
c46816_g1	up	LRR receptor-like serine/threonine-protein kinase FLS2:
c388_g2	up	Phenylalanine ammonia-lyase 1
c25197_g1	up	Anthocyanidin synthase
c46769_g3	up	Chalcone synthase 3
c42667_g2	up	UDP-glucose flavonoid 3-O-glucosyltransferase 6
c23651_g1	up	Anthocyanidin synthase
c32643_g1	up	Flavonoid 3'-monooxygenase
c42263_g2	up	Flavonoid 3'-monooxygenase
c59670_g1	up	UDP-glucose flavonoid 3-O-glucosyltransferase 7
c48323_g1	down	UDP-glycosyltransferase 86A1
c41071_g2	down	UDP-glucose flavonoid 3-O-glucosyltransferase 6
**Chlorophyll a-b binding protein**		
c25429_g1	up	Sigma factor binding protein 1, chloroplastic
c83332_g1	down	Polygalacturonase
c78439_g1	down	LHCI type II CAB
c47616_g1	down	CAB-10A
c53957_g1	down	LHCI type III CAB-P4
c59646_g1	down	LHCII protein 4.2
**Sugar accumulation**		
c46424_g1	up	Beta-glucosidase 12
c26539_g1	up	Putative alpha-L-fucosidase 1
c23588_g1	up	Bidirectional sugar transporter N3
c22022_g1	up	Lysine-rich arabinogalactan protein 18
c31609_g1	up	Sucrose synthase 6

c59597_g1	up	Bidirectional sugar transporter SWEET12
c43584_g1	up	Fasciclin-like arabinogalactan protein 1
c38453_g1	up	ABC transporter C family member 5
c11665_g1	up	MGDG synthase type A
c37376_g1	up	Glycogenin-like protein 7
c28785_g1	up	Classical arabinogalactan protein 9
c31609_g2	up	Sucrose synthase 6
c16805_g1	up	Classical arabinogalactan protein 9
c43519_g2	up	Beta-fructofuranosidase, insoluble isoenzyme CWINV1
c35815_g2	up	Putative glycosyltransferase 5
c39310_g2	up	Acetyl-CoA acetyltransferase, cytosolic 1
c46841_g1	up	Lysine histidine transporter 1
c42221_g1	down	Leucine-rich repeat extensin-like protein 3
c31627_g1	down	Galactinol synthase 1
c75967_g1	down	Organic cation/carnitine transporter 3
c39262_g3	down	Sulfated surface glycoprotein 185
c32237_g1	down	Hydroquinone glucosyltransferase
c44351_g1	down	Zeatin O-xylosyltransferase
c45793_g1	down	Lysine-specific demethylase 3B
c37451_g1	down	ABC transporter G family member 12
c59640_g1	down	ATP-dependent 6-phosphofructokinase 7;
c45588_g2	down	Probable glycosyltransferase At5g03795
c15951_g1	down	Lysine-specific demethylase 3B
c45290_g1	down	Epidermis-specific secreted glycoprotein EP1
c73045_g1	down	Hydroxyproline-rich glycoprotein 1
c60053_g1	down	Fructose-bisphosphate aldolase
c24172_g1	down	Fructose-bisphosphate aldolase cytoplasmic isozyme
**Cytochrome-related protein**		
c33311_g1	up	Cytochrome P450 CP1
c43074_g1	up	Cytochrome P450 81D11
c18476_g1	up	Cytochrome P450 CYP736A12
c66778_g1	up	Cytochrome P450 93A1
c46699_g1	up	Cytochrome P450 71D11

c40433_g1	up	Cytochrome P450 82C4
c34981_g1	up	Cytochrome P450 734A1
c78221_g1	down	Cytochrome b5 isoform B
c46125_g2	down	Full=Cytochrome c oxidase subunit 1
c53653_g1	down	Cytochrome P450 71B34
**Trace element-related protein**		
c35690_g1	up	Calmodulin-like protein 11
c37914_g1	up	Boron transporter 1
c37914_g2	up	Boron transporter 1
c30906_g1	up	Ca (2^{+})-ATPase isoform 2
c6794_g1	up	Zinc finger protein STOP1 homolog
c44870_g1	up	Potassium channel AKT1
c46335_g3	up	Homogentisate phytyltransferase 2, chloroplastic
c29896_g2	up	Calmodulin-like protein 5
c60369_g1	down	COBW domain-containing protein 1
c32683_g1	down	Zinc finger protein CONSTANS-LIKE 7
c45619_g1	down	Zinc finger protein MAGPIE
c60176_g1	down	Calcium-binding protein CML38
**Stress response**		
c59619_g1	down	Heat shock protein
c25561_g1	down	15.7 kDa Heat shock protein, peroxisomal
c46871_g2	down	Heat shock cognate 70 kDa protein
c46871_g5	down	Heat shock 70 kDa protein
c46871_g1	down	Heat shock cognate 70 kDa protein 2
c45569_g1	down	Heat shock 70 kDa protein 8
c46276_g1	down	17.3 kDa Class I heat shock protein
c46871_g3	down	Heat shock 70 kDa protein
c60423_g1	down	18.2 kDa Class I heat shock protein
c38276_g4	down	Heat shock 70 kDa protein
c41926_g1	down	Small heat shock protein, chloroplastic
c46998_g1	down	17.4 kDa Class III heat shock protein
c28107_g4	down	Heat shock cognate 70 kDa protein
c29501_g1	down	Heat shock cognate 70 kDa protein

c46276_g3	down	17.3 kDa Class I heat shock protein
c46276_g2	down	17.3 kDa Class I heat shock protein
c46567_g1	down	Heat shock protein 83
c39984_g1	down	Heat shock 40 kDa protein 1 protein 40
c32064_g1	down	23.6 kDa Heat shock protein
c36874_g1	down	Heat shock protein 82
c38276_g2	down	Heat shock 70 kDa protein 1
c31839_g1	down	Heat shock protein 90-1; Short=AtHSP90.1
c15645_g1	down	Small heat shock protein
**Dirigent protein**		
c41473_g1	up	Metalloendoproteinase 1
c40471_g1	up	Inhibitor of trypsin and Hageman factor
c25369_g1	up	Thaumatin-like protein 1
c43066_g1	up	Serine protease inhibitor 2
c35833_g2	up	Allene oxide synthase, chloroplastic
c39260_g2	up	Pathogenesis-related protein 1
c78371_g1	up	Arginine decarboxylase
c35833_g1	up	Allene oxide synthase
c36160_g1	up	Alpha-amylase/subtilisin inhibitor
c47307_g1	up	Major latex allergen Hev b 5
c35833_g3	up	Allene oxide synthase, chloroplastic
c22164_g1	up	Pathogenesis-related protein STH-2
c35850_g2	up	Subtilisin-like protease
c22521_g1	up	Pathogen-related protein
c29750_g1	up	BTB/POZ domain-containing protein At5g48130
c29646_g1	up	Dirigent protein 19
c31413_g1	up	MLO-like protein 11
c37666_g2	up	Putative disease resistance protein RGA1
c45494_g1	up	Retrovirus-related Pol polyprotein from transposon TNT 1-94
c47181_g1	up	Putative disease resistance RPP13-like protein 1
c39793_g2	up	MLO-like protein 6
c40997_g2	up	Disease resistance protein RPP13
c46256_g1	up	Isoflavone reductase-like protein

c36373_g1	up	Allene oxide synthase, chloroplastic
c17039_g1	up	Thaumatin-like protein
c47022_g4	up	Endochitinase
c73214_g1	up	Alkaline elastase YaB
c41026_g2	up	Major allergen Pru ar 1
c45198_g2	up	Endochitinase PR4
c39260_g1	up	Pathogenesis-related protein 1
c47290_g3	up	Putative disease resistance RPP13-like protein 3
c44303_g3	up	Pirin-like protein
c43392_g1	up	Inactive TPR repeat-containing thioredoxin TTL3
c8892_g1	down	Cysteine proteinase COT44
c40563_g1	down	DAG protein, chloroplastic
c28279_g1	down	Protein ASPARTIC PROTEASE IN GUARD CELL 1
c37829_g1	down	Protein EARLY RESPONSIVE TO DEHYDRATION 15
c42670_g1	down	BTB/POZ and MATH domain-containing protein 3
c47387_g1	down	Outer envelope protein 80, chloroplastic
c25738_g1	down	BTB/POZ domain-containing protein At2g30600
c42218_g1	down	Thioredoxin-like 1-1, chloroplastic
c25777_g1	down	Cysteine proteinase inhibitor 1
**Plant hormones**		
c38441_g3	up	Brassinosteroid-regulated protein BRU1
c38441_g1	up	Brassinosteroid-regulated protein BRU1
c26485_g1	up	Cytokinin dehydrogenase 3
c43993_g1	up	Cytokinin dehydrogenase 4
c24588_g1	up	Auxin transport protein BIG
c34871_g1	up	Cytokinin riboside 5'-monophosphate phosphoribohydrolase LOG7
c25300_g2	up	Auxin efflux carrier component 3
c40067_g1	up	Auxin-binding protein ABP19a
c48357_g1	up	SAUR family protein
c66500_g2	up	Auxin transporter-like protein 2
c32874_g1	up	Cytokinin riboside 5'-monophosphate phosphoribohydrolase LOG8

c36086_g2	up	9-cis-epoxycarotenoid dioxygenase NCED6, chloroplastic
c20025_g1	up	Auxin response factor 16
c2285_g1	up	Serine/threonine-protein kinase SAPK2
c1655_g1	down	Auxin-induced protein X10A
c54431_g1	down	Protein ABSCISIC ACID-INSENSITIVE 5
c45831_g4	down	Indole-3-acetic acid-induced protein ARG7
**Ethylene-synthesis pathway**		
c780_g1	up	S-adenosylmethionine synthase 1
c2984_g1	up	S-adenosylmethionine synthase 1
c33708_g1	up	S-adenosylmethionine synthase 1
c39734_g3	up	S-adenosylmethionine synthase 2
**Oxidoreductase**		
c48232_g1	up	Peroxidase 4
c17284_g1	up	Peroxidase 51
c32133_g1	up	Benzenediol:oxygen oxidoreductase 15
c1295_g1	up	Putative laccase-9c
c37320_g1	up	Benzenediol:oxygen oxidoreductase 7
c46561_g1	up	Polyphenol oxidase, chloroplastic
c40790_g1	up	Cationic peroxidase 1
c38513_g1	up	Peroxidase 15
c53614_g1	up	L-ascorbate oxidase homolog
c47328_g1	up	Tropinone reductase-like 2
c22895_g2	up	Polyphenol oxidase, chloroplastic
c45688_g4	down	Oxygen-dependent coproporphyrinogen-III oxidase, chloroplastic
c47133_g3	down	NADH-ubiquinone oxidoreductase chain 2
c45361_g3	down	Cinnamoyl-CoA reductase 1
c40391_g1	down	NuA4 complex subunit EAF3 homolog
c70747_g1	down	12-Oxophytodienoate reductase 1
c45955_g2	down	NADH-ubiquinone oxidoreductase chain 2
c78250_g1	down	1-Aminocyclopropane-1-carboxylate oxidase 1
c45694_g6	down	Putative L-ascorbate peroxidase 6
c59623_g1	down	Ribonucleoside-diphosphate reductase small chain

c32402_g2	down	Cinnamoyl-CoA reductase 1
c15969_g1	down	Glutamine synthetase nodule isozyme
**Protein kinase**		
c39208_g1	up	LRR receptor-like serine/threonine-protein kinase At1g53430
c31899_g1	up	Acyl-CoA--sterol O-acyltransferase 1
c40495_g1	up	Receptor-like protein kinase BRI1-like 3
c37679_g1	up	G-type lectin S-receptor-like serine/threonine-protein kinase RLK1
c37519_g1	up	Serine acetyltransferase 3, mitochondrial
c35789_g1	up	Polyamine transporter At3g13620
c43113_g1	up	LRR receptor-like serine/threonine-protein kinase At1g06840
c43038_g1	up	Serine/threonine-protein kinase At5g01020
c43674_g3	up	G-type lectin S-receptor-like serine/threonine-protein kinase At2g19130
c45077_g2	up	LRR receptor-like serine/threonine-protein kinase At4g36180
c2285_g1	up	Serine/threonine-protein kinase SAPK2
c40326_g1	up	Leucine-rich repeat receptor-like serine/threonine-protein kinase BAM1
c44456_g1	up	Kinesin-like protein D
c9350_g1	up	LRR receptor-like serine/threonine-protein kinase At1g56140
c20890_g1	down	Serine carboxypeptidase-like 19
c37845_g1	down	Serine/arginine-rich splicing factor SR45a
c44051_g2	down	Serine/threonine-protein kinase At1g01540
c42099_g1	down	CBL-interacting protein kinase 16
c47097_g1	down	G-type lectin S-receptor-like serine/threonine-protein kinase SD1-1
c45872_g1	down	Serine/threonine-protein kinase STN8, chloroplastic
c51272_g1	down	CBL-interacting serine/threonine-protein kinase 25
c72059_g1	down	Acyl-[acyl-carrier-protein]
c33825_g1	down	Phosphoserine aminotransferase, chloroplastic
c51376_g1	down	LRR receptor-like serine/threonine-protein kinase At4g36180
**Others**		
c41196_g1	up	(-)-Germacrene D synthase

c40756_g1	up	Prostaglandin G/H synthase 1
c23523_g1	up	MpBBI, partial
c73067_g1	up	Hypothetical protein L484_005338
c62863_g1	up	_
c47082_g1	up	FAD-binding domain-containing family protein
c41801_g1	up	_
c37714_g1	up	Carbonic anhydrase 2
c15770_g1	up	Tryptophan synthase beta chain 2
c36743_g1	up	myosin-Vb-like
c45415_g2	up	Putative mitochondrial chaperone BCS1-B
c39358_g1	up	Putative sulfate transporter 3.5
c62561_g1	up	Hypothetical protein L484_014351
c45133_g1	up	_
c27960_g1	up	Sulfate transporter 3.1
c79258_g1	up	Myosin-12
c16014_g1	up	Fatty acid desaturase 3
c17733_g1	up	Myosin-12
c45888_g1	up	Polyneuridine-aldehyde esterase-like isoform 1
c63303_g1	up	_
c22828_g1	up	BAG family molecular chaperone regulator 3
c40019_g2	up	_
c47959_g1	up	Hypothetical protein L484_022573
c22655_g1	up	Hypothetical protein L484_013383
c60796_g1	up	Unnamed protein product
c59907_g1	up	Wound-induced protein WIN2
c28345_g1	up	Hypothetical protein L484_005918
c34701_g1	up	Aquaporin TIP4-1
c44811_g1	up	LL-diaminopimelate aminotransferase
c78697_g1	up	Uncharacterized protein LOC103332309
c78290_g1	up	Auxin-induced protein 5NG4
c34941_g1	up	Hypothetical protein PRUPE_ppa015386mg
c25829_g1	up	Hypothetical protein L484_026099
c33700_g1	up	_

c41398_g2	up	Hypothetical protein L484_018234
c4888_g1	up	Hypothetical protein L484_025881
c1530_g1	up	Hypothetical protein POPTR_0001s23960g
c41682_g1	up	_
c11171_g1	up	Hypothetical protein L484_027406
c34564_g1	up	_
c33608_g1	up	Amino acid binding protein, putative
c30088_g1	up	_
c42780_g1	up	Protein spinster
c1860_g2	up	Receptor-like protein 12
c56047_g1	up	Hypothetical protein L484_020796
c66337_g1	up	_
c24015_g1	up	Hypothetical protein L484_007659
c25103_g1	up	Tryptophan aminotransferase-related protein 2
c74812_g1	up	Hypothetical protein L484_009723
c35527_g1	up	Patatin group A-3
c27620_g1	up	Polygalacturonase
c34824_g1	up	_
c79216_g1	up	Putative nitrite transporter
c27259_g1	up	Cation/H(+) antiporter 20
c68709_g1	up	Hypothetical protein L484_013008
c38792_g1	up	Cellulose synthase-like protein E1
c37873_g2	up	Uncharacterized protein LOC103332843
c45446_g2	up	Salicylate O-methyltransferase
c42600_g1	up	NAC domain-containing protein 74
c47370_g1	up	_
c67272_g1	up	_
c39584_g2	up	_
c30373_g1	up	BAG family molecular chaperone regulator 1
c15713_g1	up	Putative L-type lectin-domain containing receptor kinase S.5
c37155_g1	up	Putative protein phosphatase 2C 65
c46513_g4	up	E3 ubiquitin-protein ligase UPL1
c44135_g1	up	Hypothetical protein L484_021343

c44782_g3	up	_
c45663_g1	up	E3 ubiquitin-protein ligase ATL42
c24193_g1	up	Hypothetical protein PRUPE_ppa022164mg
c46037_g1	up	Hypothetical protein POPTR_0016s02500g
c45620_g1	up	Nucleobase-ascorbate transporter 2
c42270_g1	up	Hypothetical protein L484_012754
c58399_g1	up	_
c35483_g1	up	Hypothetical protein POPTR_0004s23990g
c78947_g1	up	Hypothetical protein L484_021563
c62717_g1	up	_
c61093_g1	up	_
c20021_g1	up	NADP-dependent malic enzyme
c26807_g1	up	_
c40307_g1	up	Hypothetical protein L484_004609
c44852_g2	up	Pleiotropic drug resistance protein 2
c44085_g2	up	Type I inositol-1,4,5-trisphosphate 5-phosphatase 11
c44761_g1	up	Hypothetical protein L484_004241
c27363_g1	up	1-Aminocyclopropane-1-carboxylate oxidase-3-like protein
c40734_g1	up	_
c32167_g1	up	Purple acid phosphatase 22
c42882_g1	up	Hypothetical protein L484_022099
c94_g1	up	_
c27378_g1	up	Hypothetical protein L484_010895
c36930_g1	up	Hypothetical protein L484_020944
c40227_g1	up	Reticulon-like protein
c43124_g2	up	Kunitz-type elastase inhibitor BrEI-like
c44823_g3	up	Wall-associated receptor kinase-like 9
c46559_g4	up	Hypothetical protein L484_016736
c66025_g1	up	UPF0496 protein [Theobroma UPF0496 protein]
c45810_g2	up	_
c61930_g1	up	NADP-dependent malic enzyme
c27130_g1	up	Sulfate transporter 3.1
c43421_g1	up	Hypothetical protein L484_017807

c37504_g1	up	Hypothetical protein L484_009496
c47362_g1	up	Hypothetical protein L484_014177
c38897_g1	up	_
c36558_g1	up	Hypothetical protein POPTR_0006s26730g
c37676_g1	up	Hypothetical protein L484_017858
c48021_g1	up	Hypothetical protein L484_018092
c66407_g1	up	Pyrophosphate-energized vacuolar membrane proton pump
c39536_g1	up	Hypothetical protein POPTR_0003s08530g
c33118_g1	up	_
c72459_g1	up	Tabersonine 16-O-methyltransferase
c59693_g1	up	Hypothetical protein L484_022042
c44617_g2	up	Probable receptor-like protein kinase At1g67000
c46901_g1	up	Ankyrin repeat-containing protein
c37460_g2	up	Phytosulfokines 2
c6715_g1	up	_
c36224_g1	up	Putative F-box/LRR-repeat protein
c46326_g2	up	Putative transporter MCH1
c44641_g1	up	Hypothetical protein L484_010028
c39639_g2	up	Hypothetical protein L484_012729
c45256_g4	up	Hypothetical protein AMTR_s00044p00088410
c60098_g1	up	Root phototropism protein 3
c22717_g1	up	Hypothetical protein L484_015854
c61608_g1	up	_
c30478_g1	up	_
c66049_g1	up	Hypothetical protein EUGRSUZ_J03081
c49238_g1	up	_
c30139_g1	up	_
c32395_g1	up	Hypothetical protein L484_019794
c42607_g2	up	Putative phospholipid-transporting ATPase 9
c24802_g1	up	Hypothetical protein PRUPE_ppa000723mg
c31425_g1	up	Hypothetical protein PRUPE_ppa009089mg
c41935_g1	up	Hypothetical protein L484_026458
c45256_g5	up	Actin

c54067_g1	up	Hypothetical protein CICLE_v10014579mg
c14492_g1	up	_
c53951_g1	up	Putative inorganic phosphate transporter 1-7
c33329_g1	up	Lysine histidine transporter 1
c34955_g3	up	Hypothetical protein L484_026266
c32935_g1	up	Hypothetical protein L484_008838
c42983_g2	up	Nitrate transporter 1.5
c34203_g2	up	_
c36701_g2	up	Hypothetical protein L484_026861
c45256_g2	up	Actin
c47117_g3	up	Oxidoreductase family protein
c27000_g1	up	_
c28074_g1	up	Hypothetical protein L484_013975
c9590_g1	up	_
c29896_g1	up	_
c17135_g1	up	Glutamate-gated kainate-type ion channel receptor subunit GluR5, putative
c26634_g1	up	DNA polymerase III subunit
c32908_g1	up	Uncharacterized protein TCM_006175
c72279_g1	up	Ribonuclease 3-like protein 3
c32488_g1	up	Hypothetical protein L484_024550
c33943_g1	up	Uncharacterized protein LOC103440565
c22654_g2	up	_
c44341_g2	up	Pectinesterase 2
c48138_g1	up	Hypothetical protein L484_005735
c44012_g1	up	Hypothetical protein L484_010090
c37653_g1	up	Glutamate dehydrogenase 2
c32060_g1	up	Hypothetical protein L484_023636
c795_g1	up	_
c3341_g1	up	_
c39525_g1	up	Hypothetical protein CICLE_v10023471mg, partial
c44788_g1	up	_
c42278_g1	up	Hypothetical protein PRUPE_ppa002180mg

c41373_g1	up	Probable receptor-like protein kinase At1g67000
c42179_g1	up	Uncharacterized protein LOC103450445
c43207_g1	up	Hypothetical protein L484_000923
c37454_g1	up	Putative carboxylesterase 2
c45272_g1	up	Hypothetical protein PRUPE_ppa015445mg
c36690_g1	up	Extracellular ribonuclease LE
c44187_g3	up	AarF domain-containing kinase isoform 2
c45836_g5	up	Hypothetical protein L484_022857
c48689_g1	up	Hypothetical protein PRUPE_ppa026773mg, partial
c15824_g1	up	Hypothetical protein L484_027304
c20244_g1	up	_
c34593_g1	up	_
c38936_g1	up	Hypothetical protein L484_004108
c4595_g1	up	Uncharacterized protein LOC100255813
c59695_g1	up	Hypothetical protein L484_013434
c42344_g1	up	Glycerol-3-phosphate acyltransferase 1
c46089_g1	up	Tabersonine 16-O-methyltransferase
c42448_g1	up	Transmembrane protein 87A
c76115_g1	up	Hypothetical protein VITISV_002159
c16772_g1	up	Hypothetical protein L484_005955
c47290_g2	up	Disease resistance protein RPM1
c32629_g1	up	Hypothetical protein L484_022781
c42536_g1	up	Hypothetical protein L484_013950
c45017_g2	up	Hypothetical protein L484_001595
c37951_g2	up	Receptor-like protein kinase HERK 1
c33015_g1	up	E3 ubiquitin-protein ligase
c34420_g1	up	Tetratricopeptide repeat-like superfamily protein
c35087_g1	up	Hypothetical protein L484_011284
c46923_g1	up	Hypothetical protein PRUPE_ppa018374mg, partial
c45901_g1	up	Phytosulfokine receptor 1
c40772_g1	up	Light-inducible protein CPRF-2, putative
c41268_g2	up	OSBP (oxysterol binding protein)-related protein 2A isoform 4
c43020_g3	up	F-box/kelch-repeat protein

c39264_g1	up	Putative peptide/nitrate transporter
c15912_g1	up	Triacylglycerol lipase SDP1
c22092_g1	up	Hypothetical protein L484_006800
c31212_g1	up	Hypothetical protein L484_005835
c47087_g1	up	TMV resistance protein N
c38432_g2	up	MATE efflux family protein LAL5
c27964_g1	up	Hypothetical protein L484_025877
c37931_g1	up	Hypothetical protein PRUPE_ppa003644mg
c40517_g1	up	Putative inactive receptor kinase
c43464_g3	up	Putative receptor protein kinase ZmPK1
c46089_g2	up	Tabersonine 16-O-methyltransferase
c46765_g1	up	Hypothetical protein PRUPE_ppa000824mg
c59705_g1	up	Blue copper protein
c25411_g1	up	Putative phosphatidylinositol 4-kinase type 2-beta
c44291_g1	up	Hypothetical protein L484_019537
c12696_g1	up	Formin-like protein 5
c45951_g1	up	Hypothetical protein PRUPE_ppa002729mg
c22373_g1	down	Hypothetical protein POPTR_0002s09970g
c22520_g1	down	Hypothetical protein L484_018717
c30528_g1	down	Hypothetical protein CICLE_v10009512mg
c45911_g1	down	_
c33938_g1	down	_
c44779_g3	down	_
c53863_g1	down	Hypothetical protein L484_010421
c15623_g2	down	_
c60212_g1	down	_
c12141_g2	down	Hypothetical protein AMTR_s00001p00156400
c38531_g1	down	Uncharacterized protein LOC100778672
c34672_g1	down	Ubiquitin-conjugating enzyme E2 28-like
c29445_g2	down	_
c44947_g2	down	Hypothetical protein L484_008155
c45314_g2	down	Ubiquitin extension protein
c23095_g1	down	Hypothetical protein L484_018408

c41524_g1	down	_
c17983_g1	down	_
c45028_g2	down	Hypothetical protein L484_002870
c23289_g1	down	40S ribosomal protein S6 isoform 1
c32818_g1	down	_
c31770_g1	down	Hypothetical protein L484_012921
c81973_g1	down	40S ribosomal protein S2-4-like
c40782_g1	down	Ras-related small GTP-binding family protein
c37067_g2	down	Hypothetical protein PHAVU_011G209600g
c72407_g1	down	Uncharacterized protein LOC103331200
c26507_g1	down	_
c43836_g10	down	Hypothetical protein L484_025703
c40758_g1	down	Hypothetical protein EUGRSUZ_C01180
c45532_g1	down	Uncharacterized G-patch domain protein DDB_G0278987-like
c79481_g1	down	_
c67147_g1	down	Hypothetical protein L484_013349
c16333_g2	down	GTPase obg
c42285_g2	down	Unnamed protein product
c43065_g1	down	Uncharacterized protein LOC103328581
c46999_g2	down	_
c32507_g1	down	_
c79119_g1	down	_
c46859_g2	down	Hypothetical protein PRUPE_ppa001084mg
c59603_g1	down	Splicing factor U2af small subunit A
c53817_g1	down	Rae1-like protein At1g80670
c46798_g5	down	Hypothetical protein L484_024429
c54229_g1	down	_
c41554_g3	down	Squalene monooxygenase
c78220_g1	down	_
c38763_g1	down	Chaperone protein
c36857_g4	down	Pyruvate kinase, cytosolic isozyme
c66450_g1	down	_
c40733_g1	down	20 kDa Chaperonin

c53572_g1	down	Hypothetical protein L484_006058
c29518_g1	down	Oxygen-evolving enhancer protein 2
c45260_g1	down	_
c43665_g1	down	Hypothetical protein L484_016056
c46346_g1	down	Putative cysteine desulfurase
c516_g1	down	_
c49063_g1	down	Hypothetical protein L484_014979
c45305_g1	down	Putative wound-induced protein
c45329_g1	down	Hypothetical protein (mitochondrion)
c78255_g1	down	F-box/kelch-repeat protein At2g44130-like isoform X1
c65852_g1	down	MutS2 protein
c45955_g8	down	Conserved hypothetical protein
c43796_g2	down	_
c5135_g1	down	Hypothetical protein L484_016856
c40137_g1	down	Hypothetical protein L484_009017
c31345_g2	down	_
c41848_g1	down	Hypothetical protein L484_023631
c30479_g1	down	_
c11941_g1	down	Hsp70-binding protein 1
c36940_g1	down	Conserved hypothetical protein
c5578_g1	down	Hypothetical protein L484_003254
c31370_g1	down	3-Ketoacyl-CoA synthase 21
c44654_g1	down	Hypothetical protein L484_004530
c66547_g1	down	_
c45436_g1	down	DNA nucleotidylexotransferase
c24707_g1	down	_
c42423_g1	down	Prolyl 4-hydroxylase subunit alpha-2
c19700_g1	down	Probable 2-aminoethanethiol dioxygenase-like
c49027_g1	down	33 kDa Ribonucleoprotein
c635_g1	down	_
c41587_g1	down	_
c39705_g1	down	_
c41386_g1	down	Ubiquitin-activating enzyme E1 1

c47334_g1	down	Uncharacterized protein LOC103319422
c32820_g1	down	Hypothetical protein PRUPE_ppa006638mg
c59768_g1	down	Mitogen-activated protein kinase kinase kinase 2
c43296_g6	down	Histone H2B [Nicotiana tabacum]
c60077_g1	down	_
c66702_g1	down	RING-H2 finger protein ATL3
c44090_g1	down	Hypothetical protein L484_004039
c34964_g1	down	Two-component response regulator
c53451_g1	down	Cyclin-dependent kinase inhibitor 7
c44815_g1	down	Putative methyltransferase NSUN5-like isoform X1
c67083_g1	down	_
c34253_g1	down	PHD finger protein ALFIN-LIKE 5
c41614_g1	down	Hypothetical protein JCGZ_25539
c37107_g1	down	Hypothetical protein L484_021058
c44321_g1	down	Hypothetical protein JCGZ_24457
c53641_g1	down	Hypothetical protein L484_024446
c9320_g1	down	Hypothetical protein L484_021185
c44555_g1	down	50S ribosomal protein L29
c45642_g1	down	Hypothetical protein L484_004416
c56769_g1	down	_
c11381_g1	down	J1P
c15791_g1	down	Hypothetical protein L484_009906
c81297_g1	down	_
c11381_g2	down	DnaJ-like protein
c33137_g1	down	Metal tolerance protein 4
c39962_g1	down	Hypothetical protein L484_002382
c13584_g1	down	DNA-directed RNA polymerase II subunit
c38023_g2	down	Ubiquitin-conjugating enzyme E2-17 kDa 10/12 family protein
c18787_g1	down	Hypothetical protein L484_020113
c29665_g1	down	Hypothetical protein L484_013163
c40571_g2	down	Hypothetical protein PRUPE_ppa000855mg
c34095_g1	down	Hypothetical protein CICLE_v10002169mg
c59680_g1	down	SODA protein

c45554_g1	down	Upstream activation factor subunit spp27-like
c37015_g6	down	_
c46767_g3	down	Peptidyl-prolyl cis-trans isomerase FKBP65
c26056_g1	down	_
c34449_g2	down	Uncharacterized protein LOC103337247
c36979_g1	down	Proliferating cell nuclear antigen large form
c22756_g1	down	Aspartokinase 2, chloroplastic isoform X2
c47131_g1	down	Germination-specific cysteine protease 1
c44648_g1	down	Protein FLUORESCENT IN BLUE LIGHT
c72587_g1	down	ATP synthase subunit alpha
c24496_g1	down	Hypothetical protein PRUPE_ppa003710mg
c66480_g1	down	Oleosin H-isoform [*Ficus pumila* var. *awkeotsang*]
c41032_g2	down	_
c40709_g1	down	_
c44815_g2	down	Hypothetical protein L484_003775
c47624_g2	down	RING-H2 finger protein ATL5
c1562_g2	down	_
c40332_g2	down	_
c40459_g3	down	_
c40269_g2	down	RNA-binding family protein, putative isoform 1
c20122_g1	down	_
c39101_g1	down	Uncharacterized protein isoform 1
c47776_g1	down	Pollen-specific leucine-rich repeat extensin-like protein 3
c78629_g1	down	Hypothetical protein L484_023221
c19212_g1	down	_
c27944_g1	down	Hypothetical protein POPTR_0007s06150g
c45642_g3	down	Putative plastid-lipid-associated protein 8
c46053_g4	down	Histone-lysine N-methyltransferase CLF
c45486_g6	down	_
c43728_g1	down	Pyrophosphate-energized vacuolar membrane proton pump 1-like
c40098_g1	down	Hypothetical protein L484_022156
c31345_g1	down	Uncharacterized protein LOC102626192

c42014_g1	down	Putative xyloglucan
c41409_g1	down	F-box/kelch-repeat protein
c43482_g2	down	Hypothetical protein L484_021141
c42848_g2	down	Hypothetical protein L484_003351
c72159_g2	down	Hypothetical protein L484_010769
c14380_g1	down	Hypothetical protein L484_020297
c53453_g1	down	Hypothetical protein L484_014149
c80552_g1	down	_
c60552_g1	down	_
c53495_g1	down	Hypothetical protein L484_012395
c42539_g3	down	Hypothetical protein L484_019883
c44390_g3	down	Uncharacterized protein LOC102631241
c47340_g1	down	GTP-binding nuclear protein Ran1-like
c44000_g2	down	Endoplasmic reticulum oxidoreductin-1-like
c37354_g1	down	Ubiquitin-activating enzyme E1 2
c40539_g1	down	_
c25334_g1	down	_
c27048_g1	down	_
c43017_g1	down	Vesicle-associated protein 4-1
c53806_g1	down	_
c26436_g1	down	Hypothetical protein L484_005181
c40427_g2	down	_
c36979_g2	down	Proliferating cell nuclear antigen large form
c37412_g1	down	_
c42386_g1	down	_
c37896_g2	down	Influenza virus NS1A-binding protein-A-like protein
c217_g1	down	Haloacid dehalogenase-like hydrolase domain-containing protein 3
c72145_g1	down	Phospholipase C 3
c35763_g1	down	Photosystem I reaction center subunit III
c38937_g1	down	Hypothetical protein PRUPE_ppa012501mg
c31216_g1	down	Uncharacterized protein LOC102628268
c41672_g2	down	Hypothetical protein L484_013065

c34195_g1	down	_
c46310_g2	down	Hypothetical protein, partial
c73293_g1	down	Uncharacterized protein TCM_029816
c11401_g1	down	Ubiquitin-activating enzyme E1 2-like
c59593_g1	down	Pyruvate decarboxylase isozyme 1
c74957_g1	down	_
c37557_g1	down	Hypothetical protein L484_003773
c36842_g1	down	_
c45702_g1	down	Hypothetical protein L484_025669
c31893_g1	down	Uncharacterized protein LOC103336598
c54501_g1	down	Hypothetical protein L484_012802
c30450_g1	down	BTB/POZ domain-containing protein
c43244_g2	down	Hypothetical protein L484_011333
c66352_g1	down	Uncharacterized protein TCM_021870
c40875_g1	down	Hypothetical protein L484_001846
c36842_g2	down	_
c45694_g9	down	Hypothetical protein L484_004489
c38692_g1	down	_
c23527_g1	down	_
c31666_g1	down	Hypothetical protein L484_013693
c61025_g1	down	Multiprotein-bridging factor 1c
c64593_g1	down	_
c37311_g1	down	Lon protease 2
c44457_g1	down	Aspartate aminotransferase, putative
c39040_g1	down	_
c43910_g1	down	BAG family molecular chaperone regulator 5
c42533_g2	down	Hypothetical protein L484_023363
c37210_g1	down	Hypothetical protein PRUPE_ppa024646mg
c46891_g3	down	Zinc finger family protein
c36467_g1	down	Chaperone protein dnaJ 6-like
c23298_g1	down	Hypothetical protein L484_017604
c47242_g1	down	Hypothetical protein
c47148_g3	down	_

c45486_g1	down	_
c12060_g1	down	Eukaryotic translation initiation factor 5A-4
c23424_g1	down	Hypothetical protein MIMGU_mgv1a011980mg
c5724_g1	down	Pleiotropic drug resistance protein 1
c78541_g1	down	Hypothetical protein L484_011513
c46227_g1	down	_
c27279_g1	down	Hypothetical protein L484_000015
c47172_g3	down	Hypothetical protein VITISV_024648
c47349_g1	down	Hypothetical protein L484_014397
c74036_g1	down	_
c47023_g1	down	tRNA (guanosine(18)-2'-O)-methyltransferase
c46209_g10	down	_
c37406_g5	down	Transcription factor
c40975_g1	down	Hypothetical protein L484_026249
c25154_g1	down	Hypothetical protein L484_004039
c38692_g2	down	_
c29538_g2	down	_
c27562_g1	down	GATA zinc finger domain-containing protein 10
c30024_g1	down	Hypothetical protein L484_003873
c27062_g1	down	_
c19765_g1	down	_
c39999_g2	down	_
c29542_g1	down	_
c25986_g2	down	14 kDa Proline-rich protein DC2.15-like
c43738_g1	down	Hypothetical protein L484_007912
c47169_g1	down	_
c45741_g2	down	Hypothetical protein L484_013397
c29873_g1	down	Hypothetical protein L484_013585
c74405_g1	down	_
c44553_g4	down	_
c53468_g3	down	Putative methyltransferase PMT21
c46749_g1	down	Uncharacterized protein LOC103323512
c30334_g1	down	Purple acid phosphatase 15

c36345_g1	down	Conserved hypothetical protein
c9875_g1	down	Hypothetical protein L484_005314
c69858_g1	down	_
c42507_g1	down	Hypothetical protein L484_026632
c47280_g1	down	Putative membrane protein
c36512_g1	down	_
c41482_g1	down	Putative peptide/nitrate transporter
c45435_g1	down	Putative uncharacterized protein
c35641_g2	down	Fibronectin-binding protein
c45943_g3	down	Alcohol dehydrogenase
c39174_g2	down	_
c43425_g2	down	Hypothetical protein Csp_D29560
c45793_g3	down	Molybdopterin biosynthesis protein CNX3
c29573_g1	down	_
c44457_g2	down	Hypothetical protein L484_016377
c45999_g2	down	Hypothetical protein PHAVU_003G241600g

FDR ≤ 0.001 and absolute value of log2 ratio ≥ 2 (2-fold) as the threshold.

*GY, young 'Green Peel' fruit; PY, young 'Purple Peel' fruit; GM, mature 'Green Peel' fruit; PM, mature 'Purple Peel' fruit.

Supplementary Figure legends

Figure S1. GO classification of unigenes of *Ficus carica* L. syconia. The results are summarized in (a) T4-P vs. T4-F; (b) T4-F vs. LD-F; (c) T4-P vs. LD-P. GO categories: Biological Process, Cellular Component and Molecular Function. T4-P, peel at stage T4; T4-F, female flower tissue at stage T4; LD-P, stage 4 peel following light-deprivation treatment; LD-F, stage 4 female flower tissue following light-deprivation treatment

Figure S1

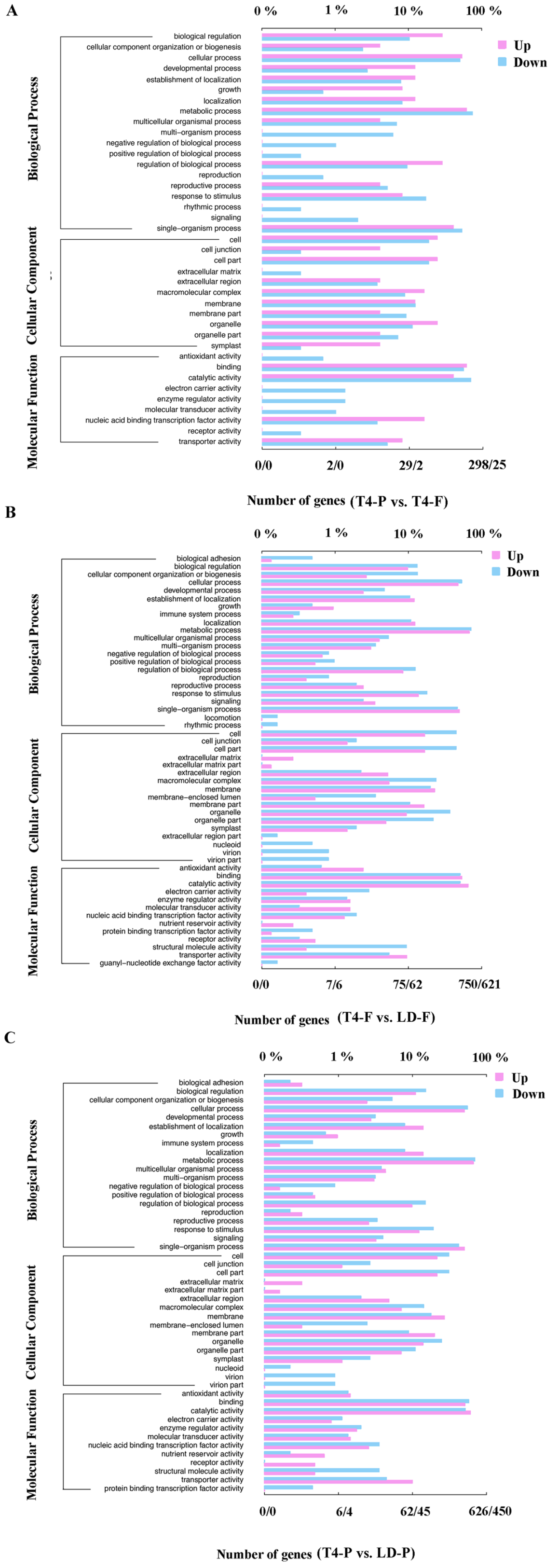
A
0 %
1 %
10 %
100 %
Up
Down
Biological Process
Cellular Component
Molecular Function
biological regulation
cellular component organization or biogenesis
cellular process
developmental process
establishment of localization
growth
localization
metabolic process
multicellular organismal process
multi−organism process
negative regulation of biological process
positive regulation of biological process
regulation of biological process
reproduction
reproductive process
response to stimulus
rhythmic process
signaling
single−organism process
cell
cell junction
cell part
extracellular matrix
extracellular region
macromolecular complex
membrane
membrane part
organelle
organelle part
symplast
antioxidant activity
binding
catalytic activity
electron carrier activity
enzyme regulator activity
molecular transducer activity
nucleic acid binding transcription factor activity
receptor activity
transporter activity
0/0
2/0
29/2
298/25
Number of genes (T4-P vs. T4-F)
B
0 %
1 %
10 %
100 %
Up
Down
Biological Process
Cellular Component
Molecular Function
biological adhesion
biological regulation
cellular component organization or biogenesis
cellular process
developmental process
establishment of localization
growth
immune system process
localization
metabolic process
multicellular organismal process
multi−organism process
negative regulation of biological process
positive regulation of biological process
regulation of biological process
reproduction
reproductive process
response to stimulus
signaling
single−organism process
locomotion
rhythmic process
cell
cell junction
cell part
extracellular matrix
extracellular matrix part
extracellular region
macromolecular complex
membrane
membrane−enclosed lumen
membrane part
organelle
organelle part
symplast
extracellular region part
nucleoid
virion
virion part
antioxidant activity
binding
catalytic activity
electron carrier activity
enzyme regulator activity
molecular transducer activity
nucleic acid binding transcription factor activity
nutrient reservoir activity
protein binding transcription factor activity
receptor activity
structural molecule activity
transporter activity
guanyl−nucleotide exchange factor activity
0/0
7/6
75/62
750/621
Number of genes (T4-F vs. LD-F)
C
0 %
1 %
10 %
100 %
Up
Down
Biological Process
Cellular Component
Molecular Function
biological adhesion
biological regulation
cellular component organization or biogenesis
cellular process
developmental process
establishment of localization
growth
immune system process
localization
metabolic process
multicellular organismal process
multi−organism process
negative regulation of biological process
positive regulation of biological process
regulation of biological process
reproduction
reproductive process
response to stimulus
signaling
single−organism process
cell
cell junction
cell part
extracellular matrix
extracellular matrix part
extracellular region
macromolecular complex
membrane
membrane−enclosed lumen
membrane part
organelle
organelle part
symplast
nucleoid
virion
virion part
antioxidant activity
binding
catalytic activity
electron carrier activity
enzyme regulator activity
molecular transducer activity
nucleic acid binding transcription factor activity
nutrient reservoir activity
receptor activity
structural molecule activity
transporter activity
protein binding transcription factor activity
0/0
6/4
62/45
626/450
Number of genes (T4-P vs. LD-P)
